# Characterization of T‐DM1‐resistant breast cancer cells

**DOI:** 10.1002/prp2.617

**Published:** 2020-06-24

**Authors:** Juliette Sauveur, Louise Conilh, Sabine Beaumel, Kamel Chettab, Lars‐Petter Jordheim, Eva‐Laure Matera, Charles Dumontet

**Affiliations:** ^1^ Cancer Research Center of Lyon INSERM 1052/CNRS 5286/University of Lyon Lyon France

**Keywords:** breast cancer, HER2, resistance, SLC transporters, T‐DM1, tubulin

## Abstract

The development of targeted therapies has drastically improved the outcome of patients with different types of cancer. T‐DM1 (trastuzumab‐emtansine) is an antibody‐drug conjugate used for the treatment of HER2‐positive breast cancer combining the FDA approved mAb (monoclonal antibody) trastuzumab and the microtubule cytotoxic agent DM1 (emtansine). Despite clinical successes achieved by targeted therapies, a large number of patients develop resistance during treatment. To explore mechanisms of resistance to T‐DM1, the MDA‐MB‐361 HER2‐positive breast cancer cell line was exposed in vitro to T‐DM1 in the absence or presence of ciclosporin A. Previously reported mechanisms of resistance such as trastuzumab‐binding alterations, MDR1 upregulation, and SLC46A3 downregulation were not observed in these models. Despite a decrease in HER2 expression at the cell surface, both resistant cell lines remained sensitive to HER2 targeted therapies such as mAbs and tyrosine kinase inhibitors. In addition, sensitivity to DNA damaging agents and topoisomerase inhibitors were unchanged. Conversely resistance to anti‐tubulin agents increased. Resistant cells displayed a decreased content of polymerized tubulin and a decreased content of βIII tubulin although the downregulation of βIII tubulin by siRNA in the parental cell line did not modified the sensitivity to T‐DM1. Both cell lines resistant to T‐DM1 also presented giant aneuploid cells. Several SLC (solute carrier) transporters were found to be differentially expressed in the resistant cells in comparison to parental cells. These results suggest that some characteristics such as increased baseline aneuploidy and altered intracellular drug trafficking might be involved in resistance to T‐DM1.

**Abbreviations**
ADCs: antibody‐drug conjugates
HER2: human epidermal growth factor receptor 2
mAb: monoclonal antibody
T‐DM1: trastuzumab‐emtansine

## 1 INTRODUCTION

Human epidermal growth factor receptor 2 (HER2) is amplified/overexpressed in approximately 20% of breast cancers and is associated with poor outcome and a high risk of recurrence.^1^, ^2^, ^3^, ^4^ Direct targeting of HER2 has dramatically improved the management of HER2‐positive breast cancer patients.^5^, ^6^ The monoclonal antibody (mAb) trastuzumab represented the first breakthrough in HER2‐targeted therapies and was followed by pertuzumab, followed by the development of antibody‐drug conjugates (ADCs).^7^, ^8^ ADCs combine the selectivity of mAbs with highly potent cytotoxic molecules, allowing to directly deliver the drug within the targeted cancer cell while reducing off‐target toxicity.^9^, ^10^ Consequently, this approach increases the therapeutic window of the drug.

Trastuzumab emtansine (T‐DM1, Kadcyla) is the first FDA‐approved ADC for the treatment of HER2‐positive metastatic breast cancer as second‐line therapy, and is also active in earlier stages of the disease.^11^, ^12^, ^13^ T‐DM1 is comprised of the monoclonal antibody trastuzumab conjugated to DM1 (emtansine), a derivative of maytansine, via a noncleavable linker (SMCC).^14^ T‐DM1 selectively binds to HER2 and delivers a potent tubulin‐binding agent within targeted cancer cells while maintaining Fc‐related antitumor activities of trastuzumab.^9^ Despite the clinical success of T‐DM1, some patients initially responding develop resistance during treatment while others present primary resistance to this agent.^15^

ADCs are relatively recent agents in the clinic and exact mechanisms of resistance to this family of agents still require in‐depth studies. However, numerous resistance mechanisms to T‐DM1 and others FDA‐approved ADCs have been highlighted so far, affecting antibody binding, ADC degradation, or conjugate availability or toxicity. T‐DM1 is prone to resistance mechanisms involving reduced trastuzumab binding, including HER2 downregulation, mutations, and masking, such as enzymatic cleavage of HER2 into p95HER2 and MUC4 masking, respectively.^16^, ^17^, ^18^ Others possible mechanisms involve the overexpression of efflux transporters such as ATP‐binding cassette (ABC) transporters.^15^, ^19^, ^20^, ^21^ More recently it has been shown that reduced intracellular release of the conjugate from the lysosomal compartment, due to reduced lysosomal proteolytic activity or altered lysosomal efflux transport, might be involved in resistance.^22^, ^23^ The solute carrier (SLC) transporter SLC46A3 is a substrate for Lys‐MCC‐DM1, the active metabolite of T‐DM1, and its downregulation is involved in T‐DM1 resistance, by limiting its cytoplasmic release.^23^ The upregulation of proteins involved in the regulation of the actin/tubulin cytoskeleton has been observed in resistance models to trastuzumab‐maytansinoid antibody‐drug conjugates.^24^, ^25^

In order to identify novel mechanisms of resistance to T‐DM1, we selected and characterized cells resistant to T‐DM1 using the MDA‐MB‐361 breast cancer cell line. To avoid the exclusive emergence of MDR‐overexpressing variants, we performed selection in the presence or absence of ciclosporin, a potent MDR inhibitor.

## 2 MATERIALS AND METHODS

### 2.1 Cell culture

The human breast adenocarcinoma cell line MDA‐MB‐361 was cultured in DMEM medium supplemented with 10% fetal calf serum and 100 µg/mL streptomycin at 37°C and 5% CO2. Cells were counted using a Cellometer Auto T4 (Nexcelom Bioscience LLC).

Selection of TR and TCR cells was performed by exposure to increasing concentrations of T‐DM1 for 6 months. Ciclosporin A (CsA, C3662; Sigma‐Aldrich) at 1 µg/mL was also added at the same time as T‐DM1 for the selection of the TCR cell line.

### 2.2 Cytotoxicity assays

Cells were seeded in 96‐well plates at a density of 8000 cells per well and incubated overnight. Increasing concentrations of chemotherapy agents were added to the media and 6 days later, viability was determined by the MTT (3‐(4,5‐dimethylthiazol‐2‐yl)‐2,5‐diphenyl tetrazolium bromide) assay. 20 µL of a 5 mg/mL MTT solution was added to each well and plates were incubated at 37°C for 4 hours. The media/MTT mix were removed and 100 µL of 4% HCl 1N/isopropanol per well were added to dissolve the purple formazan crystals. The absorbance was measured at 570 nm with 690 nm as a reference readout using a Thermo MultiSkan EX microplate reader. The absorbance of drug‐exposed and control cells was compared to determine the percentage of living cells. IC50 values were calculated using CompuSyn and Graphpad Prism softwares.

### 2.3 Real‐time cell analysis

The xCELLigence RTCA DP instrument (ACEA Bioscience) monitors cell impedance in real time. Cells were inoculated in E‐plate 16 at a density of 10 000 cells per well and incubated overnight before the addition of cytotoxic agents. The cell index was monitored for one week.

### 2.4 Efflux assays

Cell suspension was prepared with 4^e^6 cells in 10 mL of DMEM media containing 0.5 µg/mL Rhodamine 123 (Santa Cruz, sc‐208306) and incubated for 30 minutes at 37°C, 5% CO_2_. Cells were washed three times in cold DPBS on ice and some cells were taken for flow cytometry analysis (“uptake”). The remaining cells were incubated in DMEM media in the absence or presence of 3 µg/mL of CsA and incubated for 24 hours. Cells were suspended using trypsin and washed on ice before flow cytometry analysis.^26^

### 2.5 Flow cytometry analyses

Cells were incubated for 30 min at room temperature with the corresponding antibodies: HER2 (4 225 666), BCRP1 (561 180) from BD Bioscience, MDR1 (348 608) from Biolegend or mouse IgG1 κ control isotypes from BD Pharmingen. Analysis was performed using a BD LSRII flow cytometer with BD FACSDiva software (BD Biosciences) and FlowJo software (Tree Star).

### 2.6 Annexin‐X/propidium iodide apoptosis assay

Cells were seeded in 6 well plates at a density of 2^e^5 cells per well and incubated overnight. Then cells were exposed to T‐DM1 for 72 hours. Cells were harvested, washed with cold DPBS containing 10% SVF and stained using Annexin‐V‐FLUOS Staining Kit (Roche) according to the manufacturer's protocol. Analysis was performed by flow cytometry. Annexin‐V‐positive cells exposed to T‐DM1 were normalized to a control for each cell line.

### 2.7 Cell cycle distribution analyses

Cells were seeded as described for the apoptosis assay, incubated and exposed to 100 nmol/L T‐DM1 for 24 hours. Cells were harvested, washed with cold DPBS and incubated for 30 minutes at 4°C with propidium iodide (0.05 mg/mL) containing Nonidet‐P40 (0.05%) and 4 µmol/L of trisodium citrate. Cells were filtered using Falcon tubes with cell‐strainer cap (352 235) before flow cytometry analysis.

### 2.8 Western blots

Protein extraction was performed using complete RIPA buffer (RIPA buffer, 1 mmol/L DTT, 1M NaF, 100 mmol/L sodium orthovanadate, phosphatase inhibitor buffer and protease inhibitors). After SDS PAGE separation, proteins were transferred onto a PVDF membrane by iBlot dry blotting system (Invitrogen). Membranes were incubated overnight at 4°C with primary antibodies and 1 hour at room temperature with secondary antibodies (IRDye Infrared Dyes from LI‐COR Biosciences). Primary antibodies used were as follows: HER2 (GTX50425; Genetex), βIII‐tubulin (clone TUJ1), βII‐tubulin (clone 7B9), α‐tubulin (T6199), β‐tubulin (T4026), and β‐actin (A5441) from Sigma‐Aldrich. Membranes were scanned using Odyssey infrared imaging system (LI‐COR Biosciences) and densitometric quantification was performed with Odyssey software. Expression levels of proteins were normalized against β‐actin.

### 2.9 Separation of soluble tubulin and microtubules

20 ^e^6 cells were lysed in 300 µL of PEM 50DP Buffer (50 mmol/L Pipes, 1 mmol/L EGTA, 1 mmol/L MgSO_4_, 0.05% sodium azide, 1 mmol/L DTT, and proteinase inhibitors at pH 6.7) by three freeze‐thaw cycles. Cells were ultracentrifuged (100 000 g for 1 hour at 20°C) to separate soluble tubulin (supernatant) and microtubules (pellet). The pellet was suspended in 100 µL of PEM 50DP buffer, incubated on ice for 30 minutes to depolymerize tubulin and ultracentrifuged at 50 000 g for 45 minutes at 4°C to recover the supernatant. The supernatant was incubated with 1 mmol/L GTP for polymerization at 35°C for 30 minutes and ultracentrifuged at 50 000 g for 45 minutes at 35°C. After centrifugation, the supernatant was discarded and the pellet containing was suspended in 50 µL of PEM 50DP buffer. Tubulin amount in both fractions was determined by Western blot.

### 2.10 RT‐qPCR

RNA extraction was performed with the QIAamp RNeasy Mini Kit (QIAGEN) and followed by reverse transcription. Primers were designed according to Roche sequences and the NCBI primer‐BLAST designer (Table S1) and quantitative PCR analysis were performed with the LightCycler 480 Real‐Time PCR system (Roche Life Science). Gene expression was normalized using ribosomal 28S as the house‐keeping gene.

### 2.11 Statistical analyses

Each experiment was performed at least three times and the results were presented in graphs as the mean ± SD. Graphs and statistical analyses were performed using GraphPad Prism software. Statistics on cell survival experiments such as AnnexinV/PI staining or MTT assay were performed by two‐way ANOVA followed by a Bonferroni posttest. Statistics on gene expression by RT‐qPCR and fold calculation in MTT assays were performed with the Student's *t* test.

### 2.12 Materials

T‐DM1 and S‐methyl DM1 were kindly provided by Roche and ImmunoGen, respectively. Pertuzumab and cisplatin were purchased from Mylan. Trastuzumab was purchased from Virbac. Afatinib, vinorelbine, and lapatinib were purchased from Vidal. Fluorouracil and doxorubicin were purchased from Accord Healthcare. DM1 (emtansine) and colchicine were purchased from Abcam and Sigma, respectively. Paclitaxel and vincristine were purchased from Bristol Myers and Teva, respectively. Irinotecan was purchased from Hospira. PNU‐159682 was kindly provided by Mablink Bioscience.

## 3 RESULTS

### 3.1 In vitro generation of MDA‐MB‐361 models resistant to T‐DM1

MDA‐MB‐361‐resistant cells were selected in vitro by constant exposure to increasing concentrations of T‐DM1. The initial concentration of T‐DM1 was 20% of the IC50 measured after a 72‐hour exposure and was gradually increased. The final concentration of T‐DM1 reached 0.4 nmol/L, which corresponds to two times the initial IC50. Cell line selection was performed in the absence or presence of ciclosporin, a modulator of MDR1, a member of the ABC transporter family, as this transporter has been reported to perform efflux of DM1 outside the cells.^27^, ^28^ Consequently, ciclosporin A (CsA) was used to inhibit MDR1 and avoid increased efflux activity. Two cell lines resistant to T‐DM1 were therefore selected in the absence (MDA‐MB‐361 TR) or in the presence of CsA (MDA‐MB‐361 TCR) and compared to the parental cell line (MDA‐MB‐361 S).

### 3.2 Sensitivity to anti‐cancer agents

**FIGURE 1:**
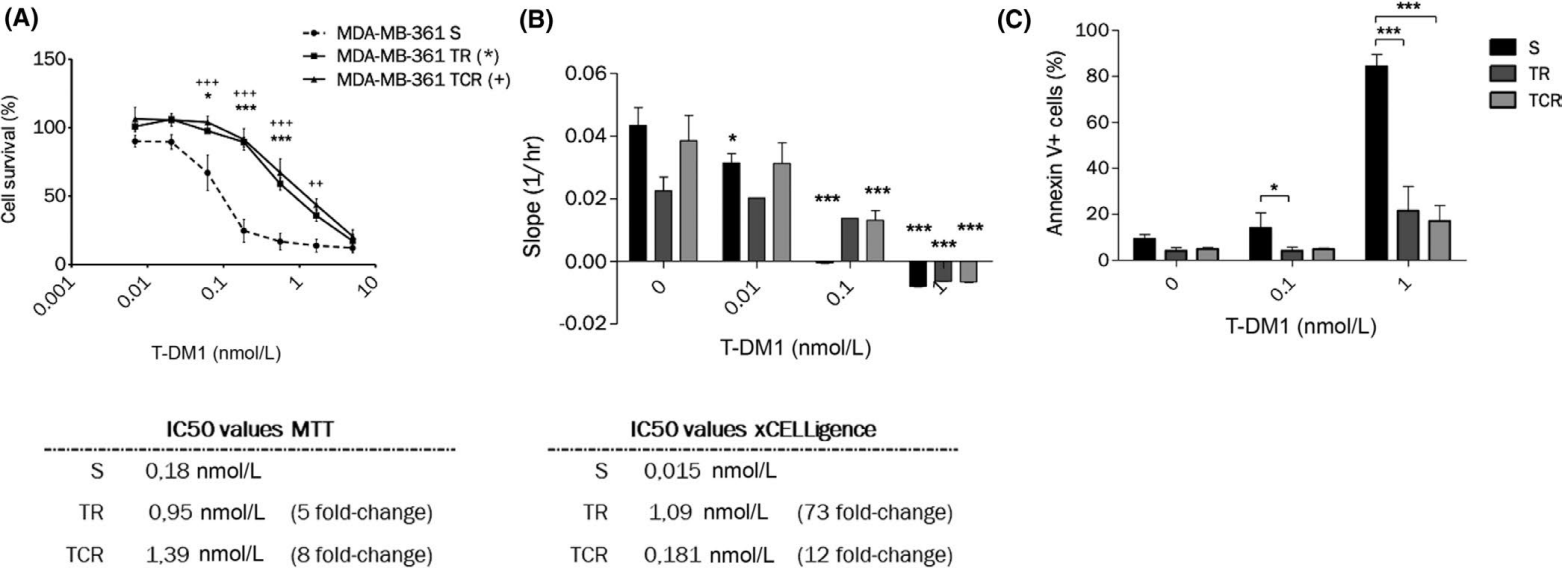
Chronic exposure to T‐DM1 of MDA‐MB‐361 cell line results in decreased sensitivity to the ADC. (A) MTT cytotoxic assays of T‐DM1 on MDA‐MB‐361 S, TR and TCR show an increase in the IC50 values of both resistant cells compared to parental. Statistical analysis was performed by two‐way ANOVA followed by Bonferroni posttests and differences are shown for TR (***: *P* < .001; **: *P* < .01; *: *P* < .05) and TCR (+) compared to S. (B) Parental and resistant cells were exposed to increasing concentrations of T‐DM1 and the cell index was followed by xCELLigence. The slopes of the normalized cell index determined by the RTCA software were plotted. Statistical analysis was performed by Two‐way ANOVA followed by Bonferroni posttests and differences are shown for each cell line between control and exposed conditions (*: *P* < .05; ***: *P* < .001). (C) Annexin‐positive cells were studied by flow cytometry after a 6‐day exposure to T‐DM1. The percentage of Annexin‐positive cells decreased in TR and TCR compared to parental cells. Statistical analysis was performed by two‐way ANOVA followed by Bonferroni posttest (*: *P* < .05; ***: *P* < .001)

Regarding resistance to T‐DM1 the IC50 determined by MTT assay was increased by fivefold in the TR cell line and by eightfold in the TCR cell line when compared to the parental cell line (Figure 1A). The IC50 calculated by xCELLigence was also increased in TR cells by 73‐fold and TCR cells by 12‐fold compared to S cells (Figure 1B). Apoptosis was analyzed by Annexin V staining after exposure to T‐DM1 for 6 days and a decreased sensitivity to T‐DM1‐induced apoptosis in TR and TCR cells was observed, compared to S cells (Figure 1C). Altogether, these results indicate that the selected TR and TCR cell lines are resistant to T‐DM1.

To assess the sensitivity of parental and resistant cell lines to various anti‐cancer agents, cytotoxicity assays were performed using targeted therapies and chemotherapy agents (Table 1). We did not observe altered sensitivity to HER2‐targeting agents, including the two FDA‐approved antibodies pertuzumab and trastuzumab nor against the tyrosine kinase inhibitors lapatinib while the TR line displayed low‐level resistance to afatinib. Both cell lines resistant to T‐DM1 remained sensitive to DNA damaging agents and topoisomerase I inhibitors, indicating that DNA‐repairing machinery may not be involved in resistance to T‐DM1. Both cells developed resistance to tubulin‐binding agents such as paclitaxel, vincristine, vinorelbine, colchicine, DM1, and S‐methyl DM1.

**TABLE 1:** Sensitivity to HER2‐targeted therapies and standard‐care chemotherapeutics

			MDA‐MB‐361 S	MDA‐MB‐361 TR		MDA‐MB‐361 TCR	
Drug target		Drug name	IC50 (nmol/L)	IC50 (nmol/L)	Relative resistance	IC50 (nmol/L)	Relative resistance
HER2		T‐DM1	0.23	1.95	7.9^(***)^	1.35	5.3^(*)^
		Trastuzumab^(#)^	0.11	0.16	**1.4**	0.21	**1.9**
		Pertuzumab	9.8	18.7	**1.9**	12.31	**1.3**
		Lapatinib	4817	4428	**0.9**	3227	**0.7**
		Afatinib	23.2	43.7	2.4(*)	13.1	**0.6**
Topoisomerase I		Irinotecan	3443	5203	**1.5**	3797	**1.1**
		PNU‐159682	0.10	0.16	**1.0**	0.1	**0.8**
DNA	Antimetabolite	Fluorouracil	16 227	9020	**0.6**	15 323	**0.9**
	Alkylating	Cisplatin	3867	7023	**1.8**	9457	**2.4**
	Intercalating	Doxorubicin	6.57	7.31	**1.1**	8.81	**1.3**
Tubulin	Polymerizing	Paclitaxel	0.08	0.17	**2.0**	0.23	**2.7**
	Depolymerizing	Vincristine	0.12	0.40	**3.2**	0.67	5.3^(*)^
		Vinorelbine	2.26	3.99	2.8^(*)^	4.61	**4.8**
		Colchicine	1.98	3.84	1.8^(*)^	2.27	**1.5**
		S‐methylDM1	0.07	0.16	**2.3**	0.13	**1.8**
		DM1	1.68	4.40	2.9^(*)^	3.52	**4.7**

Note

The sensitivity after a 6 day exposure to the indicated anti‐cancer agents was studied by MTT cytotoxic assays. Data shown as the IC50 values of a single experiment, representative of 3‐4 independent experiments, and the relative resistance is the mean ratio of the IC50 of each resistant cell line over the parental cell line from 3‐4 independent experiments (#: IC75). Statistics were calculated using the Student *t* test (*:*P* < .05; **P < .01; ***P < .001).

Bold values indicate relative resistance numbers

### 3.3 HER2 expression is decreased in cells resistant to T‐DM1

**FIGURE 2:**
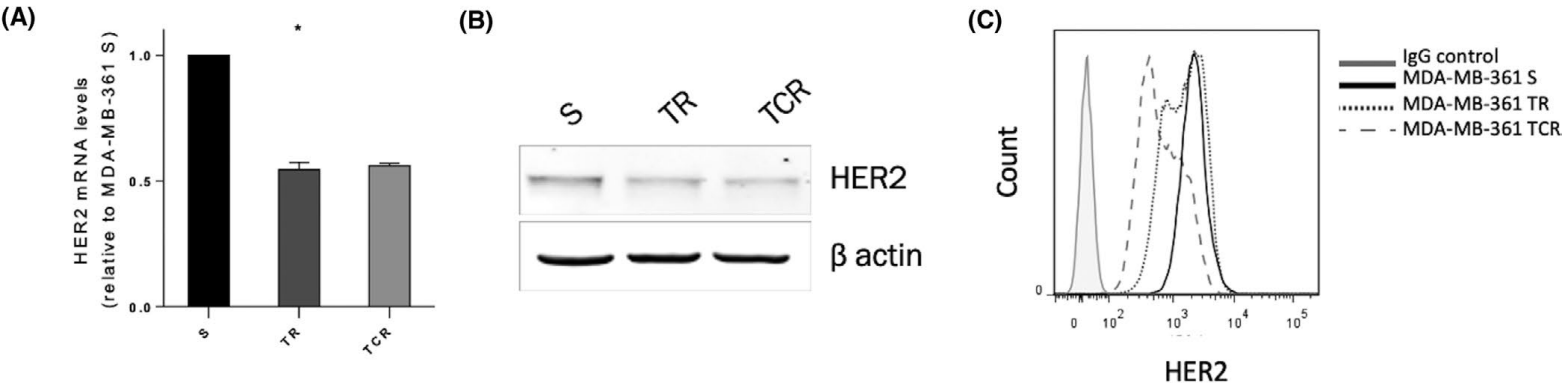
HER2 expression is decreased in resistant cells to T‐DM1. (A) mRNA and (B) protein expression from total cell lysates show that HER2 levels are decreased in resistant cells. Statistics were calculated using the Student's *t* test (*: *P* < .05). (C) HER2 expression at the cell surface determined by flow cytometry shows distinct populations in resistant cells expressing HER2^high^ and HER2^low^, especially in TCR cells. The total mean fluorescence intensity of HER2 is decreased in resistant cells compared to parental ones

HER2 expression at the cell surface is required for T‐DM1 activity. Therefore, its expression was investigated by RT‐qPCR, western blot, and flow cytometry. A downregulation of HER2 was observed at the mRNA and the protein levels in both resistant cell lines compared to the parental cell line (Figure 2A‐C). Surprisingly, a heterogeneous population expressing HER2^high^ and HER2^low^ in TR and TCR cell types was identified. This indicates that during the prolonged exposure to T‐DM1 a subpopulation of cells expressing low levels of HER2 was selected, with presumably reduced sensitivity to the cytotoxic agent.

### 3.4 Efflux activity is increased in resistant models

**FIGURE 3:**
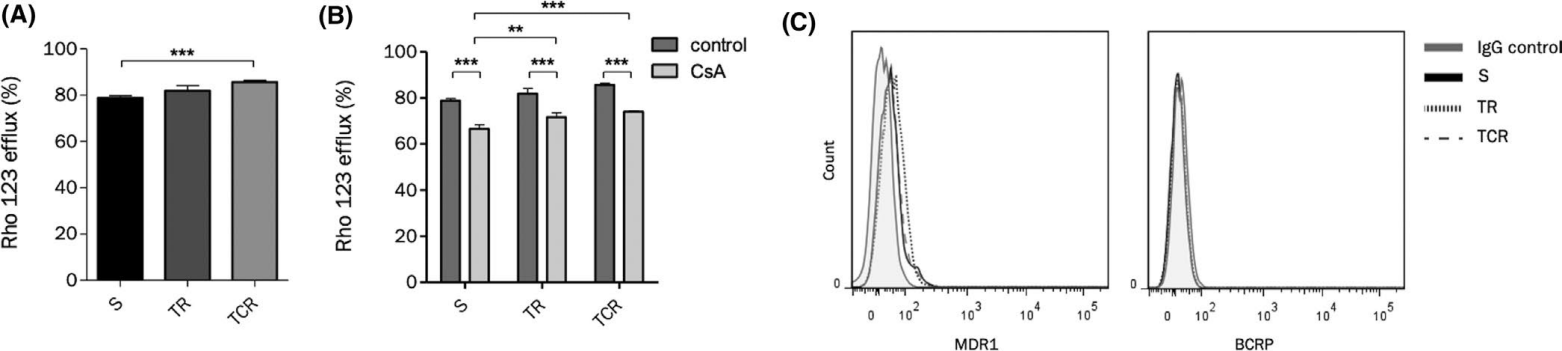
Efflux activity is increased in TCR cells but MDR1 and BCRP expression remain unchanged. (A) Efflux activity was studied by accumulation of rhodamine 123 (Rho 123) using flow cytometry. The efflux activity is increased only in TCR cells compared to parental cells. (B) Efflux activity was studied in the absence or presence of CsA, an inhibitor of MDR1. The addition of CsA reduces the Rho123 efflux percentage in both parental and resistant cell lines. (C) The expression of MDR1 and BCRP detected by flow cytometry indicates that resistant cell lines do not overexpress either ABC transporter

Overexpression of ABC transporters is a common mechanism of multidrug resistance.^29^, ^30^, ^31^ To verify whether resistance to T‐DM1 was due to the enhanced expression of ABC transporters, a rhodamine 123 (Rho 123) accumulation assay was performed (Figure 3A). Increased efflux activity was observed in TCR cells compared to parental cells, while it remained unchanged in TR cells. To determine whether this increase in efflux activity was due to the overexpression of proteins sensitive to modulation by CsA, Rho 123 efflux was studied in the presence of CsA (Figure 3B). CsA was found to induce a decrease in Rho123 efflux in the parental and resistant lines, suggesting that MDR1 and BCRP, the two main CsA‐sensitive efflux pumps are equally expressed in the three models.^32^, ^33^ Flow cytometry data confirmed this observation (Figure 3C).

However, Rho 123 efflux remained significantly higher in the TR and TCR cell lines than in the S cell line in presence or absence of CsA, suggesting that overexpression of a CsA‐insensitive efflux pump might significantly contribute to T‐DM1 resistance. MRP1 and MVP/LRP (major vault protein/lung resistance protein) are also associated with the MDR efflux pump family,^34^, ^35^, ^36^ and may be less sensitive to CsA inhibition. In addition, MRP1 has been reported to be overexpressed in a trastuzumab‐maytansinoid antibody‐drug conjugate‐resistant model.^20^ Analysis of MVP/LRP in the sensitive and resistant lines did not show an increased content (Figure S1).

### 3.5 SLC gene expression is altered in resistant cell lines

**FIGURE 4:**
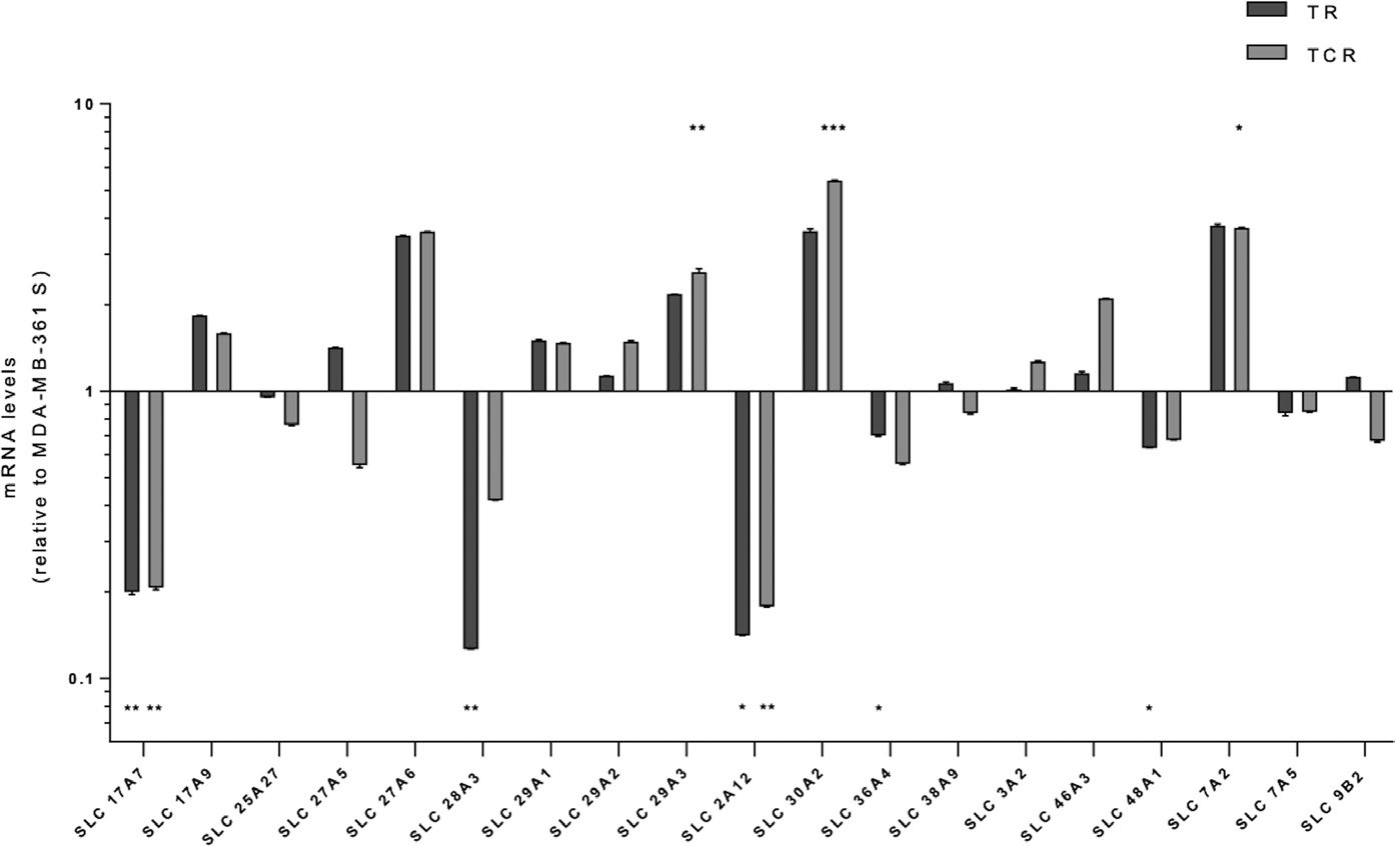
SLC transporter gene expression. The expression levels of selected SLC transporters were assessed by RT‐qPCR. mRNA levels were normalized to the 28S gene and then compared to the parental cell line (MDA‐MB‐361 S) as the fold of each resistant cell line over the parental one. Data are shown as the fold of a single experiment, representative of three independent experiments. Statistics were calculated using the Student's *t* test (*: *P* < .05; **: *P* < .01; ***: *P* < .001)

Solute carrier (SLC) transporters are involved in the transport of a wide range of substrates across membranes. Particularly, SLC46A3 has been found to allow the cytoplasmic release of Lys‐MCC‐DM1 from lysosomes. RNAseq data have shown expression changes in 52 SLCs (data not shown) among which 17 are presumably located in endosomes and/or lysosomes (Table S1). Their expression was assessed by RT‐qPCR in parental and resistant cell lines (Figure 4). Most of the results are in line with RNAseq data (Table S1), showing a downregulation of seven SLCs (SLC17A7, SLC25A27, SLC28A3, SLC2A12, SLC36A4, SLC48A1, and SLC7A5), and upregulation of eight SLCs (SLC17A9, SLC27A6, SLC29A1, SLC29A2, SLC29A3, SLC30A2, SLC46A3, and SLC7A2) in both resistant cell lines compared to the parental one. In total, seven of these have shown a strong difference in expression in TR and TCR, respectively, compared to the parental cell line: SLC17A7 (5.0 and 4.8 times fold), SLC27A6 (3.5 and 3.6 times fold), SLC28A3 (7.9 and 2.4 times fold), SLC29A3 (2.2 and 2.6 times fold), SLC2A12 (7.0 and 5.6 times fold), SLC30A2 (3.6 and 5.4 times fold), and SLC7A2 (3.7 and 3.7 times fold). Unexpectedly, SLC46A3 gene was slightly upregulated in both resistant cell lines.

### 3.6 Tubulin βIII expression and polymerized tubulin fraction were decreased in resistant models

**FIGURE 5:**
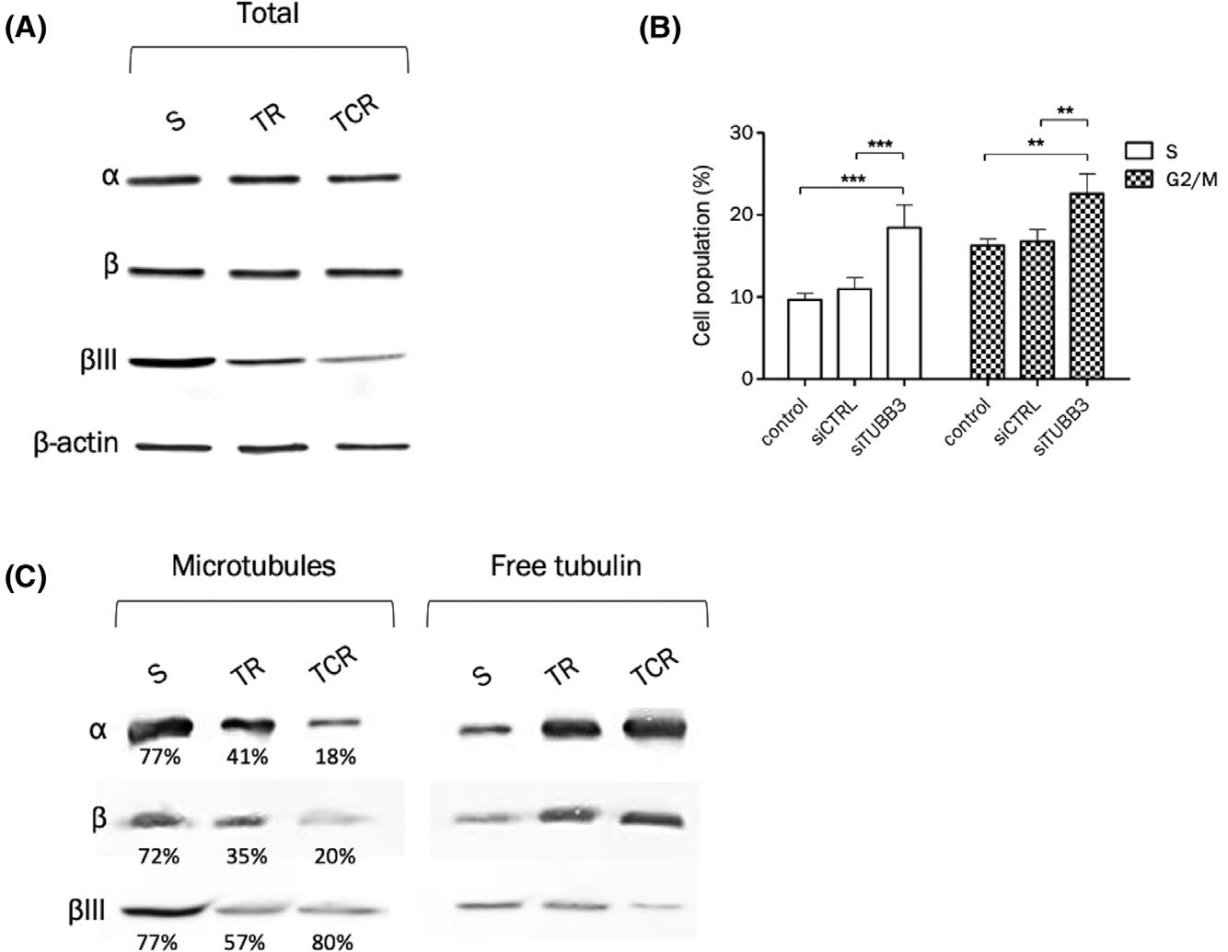
Decreased tubulin βIII expression is associated with decreased polymerized tubulin fraction and increased S and G2/M phase fractions. The protein expression of total α and β tubulin and isoform βIII was studied in total cell lysates (A) or after purification of tubulin fractions (C). (A) Western blot of tubulins α, β, and βIII shows that while total α and β tubulin expression are unchanged, βIII protein expression is decreased in T‐DM1‐resistant cells. The density of each band was normalized to actin. (B) Downregulation of βIII tubulin in the parental cell line leads to increased S and G2/M populations, 48 hour after transfection by siRNA. (C) Tubulin purification was performed to separate the polymerized (microtubules) and soluble (free) tubulin fractions. The percentage values correspond to the amount of polymerized tubulin in each cell line, for each tubulin type. The percentage of total α and β tubulin in microtubules is decreased in resistant cell lines compared to the parental one. The percentage of βIII tubulin in microtubules is decreased in the TR cell line. Even though the percentage of βIII tubulin in microtubules is unchanged in TCR cells compared to parental cells, the density of the bands indicates a higher amount of βIII tubulin in parental than resistant cells in the microtubule fraction

The microtubule/tubulin complex is the major intracellular target of T‐DM1 after the release of the active metabolite Lys‐MCC‐DM1 into the cytoplasm. The expression of total α and β tubulin was assessed by Western blot and results showed unchanged expression in TR and TCR cells compared to the parental cell line, while total βIII tubulin isotype was downregulated in TR and TCR cells (Figure 5A). To determine a possible causative relationship between βIII tubulin expression and sensitivity to T‐DM1, the MDA‐MB‐361 S cell line was transfected with a siTUBB3 or a control siRNA. The downregulation of βIII tubulin did not impact the sensitivity to T‐DM1 in parental cells (data not shown), although the populations in S and G2/M phases were increased in parental cells transfected with siTUBB3 (Figure 5B).

The amount of polymerized tubulin was studied after separation of soluble tubulin and microtubules (Figure 5C). Total α and β tubulin contained in microtubules were decreased in both resistant cell lines, and more markedly in TCR cells. Approximately 72%‐77% tubulin was found in the microtubule fraction of parental cells and only 35%‐41% in TR cells and 18%‐20% in TCR cells. The amount of βIII tubulin contained in microtubules in the parental cell line was higher than in both resistant cell lines.

Overall these results suggest that T‐DM1‐resistant variants display complex modifications of their microtubule/tubulin complex. The contribution to resistance is likely to be multifactorial since alteration of a single parameter such as tubulin III beta content was not sufficient to reproduce the resistance phenotype.

### 3.7 T‐DM1‐induced cell cycle arrest is maintained in resistant cell lines

**FIGURE 6:**
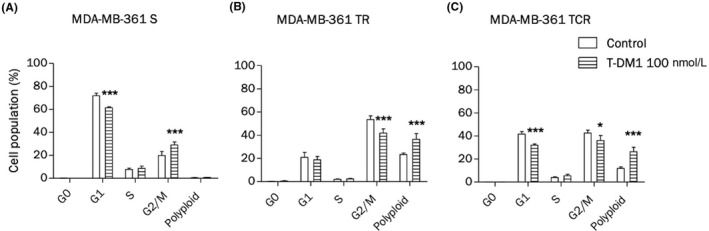
T‐DM1‐induced cell cycle arrest is maintained in resistant cells. Cells were exposed to T‐DM1 for 48 hour and cell cycle distribution was analyzed by propidium iodide staining using flow cytometry. (A) T‐DM1 induces G2/M phase arrest in the parental cell line. (B and C**)** The G1 population decreases while the G2 population increases in resistant cell lines compared to the parental line

Cell cycle distribution after a 24‐hour exposure to T‐DM1 was studied in parental and resistant cells by propidium iodide staining and flow cytometry. MDA‐MB‐361 S cells were arrested in G2/M phase after exposure to T‐DM1 (Figure 6A). Results indicate that the G1 diploid population in nonexposed MDA‐MB‐361 TR and TCR cells was decreased in comparison to S cells, whereas G2/M and polyploid populations were increased (Figure 6), suggesting that resistant cells contain an abnormally increased number of chromosomes. A decrease in the diploid G1 phase in TCR cells was observed after exposure to T‐DM1, while the amount of polyploid cells increased in both resistant cell lines, indicating that resistant cells remained sensitive to T‐DM1‐induced cell cycle arrest.

### 3.8 T‐DM1 resistant cells are giant and aneuploid

**FIGURE 7:**
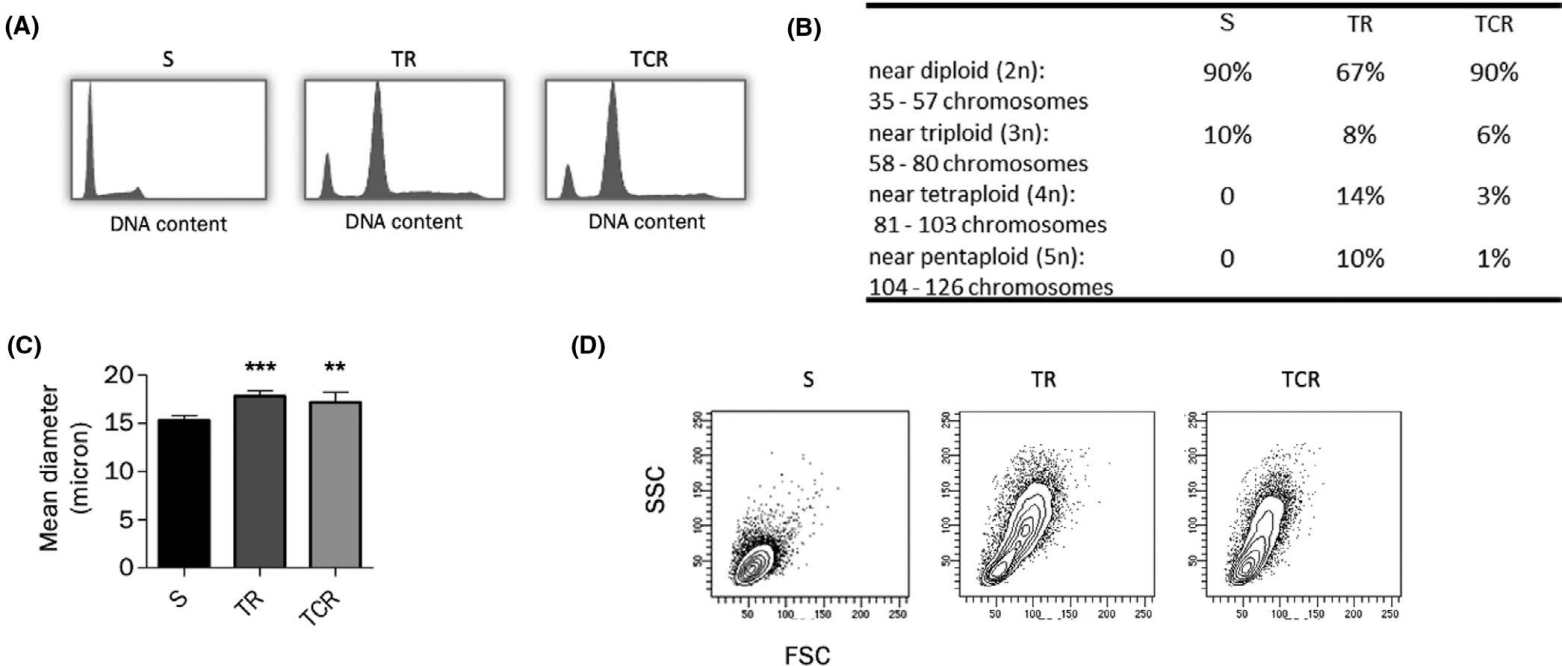
Cells resistant to T‐DM1 are giant aneuploid cells. (A) The cell cycle distribution profile was studied by propidium iodide staining in confluent cells without exposure to any agent. The G2/M fraction is increased and cells containing n > 2 appear in resistant cell lines. (B) Chromosome counts show an increase in 3n, 4n, and 5n cells in resistant cell lines, especially in MDA‐MB‐361 TR. (C) The mean diameter of cells in suspension was measured using a Cellometer counter. Cell size is increased in MDA‐MB‐361 TR and TCR cell lines in comparison to MDA‐MB‐361 S. (D) The FSC (relative size) and SSC (relative granularity) parameters determined by flow cytometry indicate a different size and granularity of cells in the TR and TCR populations compared to the S cells

To confirm the differences in cell cycle distribution observed in resistant cells, cell line DNA profiles were investigated by propidium iodide staining and flow cytometry in confluent cells (Figure 7A). The number of tetraploid (4N) and aneuploid (>4N) cells in TR and TCR cell lines was increased in comparison to parental cells. These results lead us to evaluate the ploidy in parental and resistant cells by chromosome counting (Figure 7B). The parental cell line contained 90% near diploid cells and 10% near triploid cells, whereas the number of diploid cells decreased in TR cells and remained unchanged in TCR cells. The number of 4N and 5N cells increased in both resistant cells lines, confirming the results obtained by flow cytometry. Also, as resistant cells seemed to be larger than parental cells, their mean diameter was measured by a Cellometer cell counter, as well as their SSC‐FSC parameters by flow cytometry. Data showed an increased size in TR and TCR cells compared to parental cells (Figure 7C and D). In addition, several genes involved in cell adhesion have been found to be differentially expressed in the three models (Figure S2). Altogether, these results indicate that resistant cell lines contain giant aneuploid cells with possibly altered adhesion behavior.

## 4 DISCUSSION

Clinical studies have demonstrated that T‐DM1 is prone to lose its benefit in some patients despite continuous treatment.^37^, ^38^ We selected in vitro resistant models to T‐DM1 using MDA‐MB‐361 breast cancer cells to characterize alterations occurring in resistant variants and possible mechanisms of resistance, some of which have not yet been reported in the literature. This study was conducted using a single cell line with the generation of two T‐DM1‐resistant variants, suggesting that these observations should be confirmed on other resistant models.

The downregulation of HER2 at the cell surface may interfere with T‐DM1 cytotoxicity in the selected cell lines. However, TR and TCR populations are heterogeneous for HER2 expression since they still contain HER2^high^ expressing cells that were resistant to T‐DM1. This observation is similar to that of Chen et al. who reported heterogeneity in CD20 expression in Karpas‐299 cells resistant to brentuximab vedotin.^19^ In addition, resistant cells maintained sensitivity to mAbs targeting HER2 and lapatinib suggesting that HER2 binding and accessibility were not a major mechanism of resistance in spite of its decreased expression. The cross‐resistance phenotype showed cross‐resistance to various tubulin‐binding agents, possibly in relationship with microtubular alterations. A significant level of resistance to vinorelbine was observed, which could be associated with abnormal expression of βIII tubulin.^39^ However, a decrease in tubulin beta‐III content by siRNA was not sufficient to reproduce the resistance phenotype. In spite of a slightly increased efflux activity, we did not observe cross‐resistance to other agents such as doxorubicin, suggesting that the increased efflux was not sufficient to protect cells against the toxicity of these molecules.

Both resistant cell lines remained sensitive to T‐DM1‐induced cell cycle arrest, while increased fractions of aneuploid cells were observed in the TR and TCR cell lines both at baseline and after exposure to T‐DM1. Additionally, resistant cells were larger than parental cells. These data suggest that endoreduplication is more common in resistant cells than in parental cells. Aneuploidy is associated with chromosomal instability, an increase in gene transcription, heterogeneity and adaptability, thus potentially favoring subpopulation evolution and resistance to treatment.^40^, ^41^ Correlations between aneuploidy and resistance to anti‐cancer agents have been described in previous studies suggesting that the aneuploid character of both TR and TCR cell lines compared to the sensitive one is potentially involved in the resistance to T‐DM1.^42^, ^43^, ^44^

The solute carrier (SLC) transporter family includes 418 members, divided into 52 families. The substrates for these transporters are highly diverse and some of the families are known to interact specifically with a type of substrate such as organic anions and cations (SLC22 or SLCO, respectively), nucleosides (SLC28A and SLC29A), oligopeptides (SLC15A),^45^ and amino acids (SLC1, SLC3, SLC6, SLC7, SCL25, and SLC36).^46^ Also, some SLC families have been reported to confer sensitivity and resistance to cytotoxic drugs (SL21, SLC22, SLC47, SLC31, SLC15, SLC7A, SLC38A2).^47^ SLC transporters mainly allow their substrates to cross the cell membrane. However, as some of them are located in endosomes or lysosomes, they could be responsible for nonmembrane permeable drug transport across the endosomal or lysosomal membrane. Thus, a variable expression of these SLC may affect the ADC’s active metabolite release and therefore reduce its intracellular concentration. SLC46A3 is a substrate for noncleavable ADCs metabolites such as Lys‐MCC‐DM1, the active metabolite of T‐DM1 and it has been shown that a downregulation of its expression is a mechanism of resistance to T‐DM1.^15^, ^48^

In our resistant variants, significant modifications of expression of four SLCs presumably located in the endosome and/or lysosome (SLC27A6, SLC29A3, SLC2A12, and SLC30A2) may affect lysosomal trafficking, degradation or release of Lys‐MCC‐DM1. ZnT2 (encoded by SLC30A2) is a zinc transporter from the cytoplasm into lysosomes.^49^ The upregulation of this transporter may induce zinc accumulation into the lysosome and trigger lysosomal dysfunction.^50^ Other SLC transporters presumably located in lysosomes or endosomes were found to be differentially expressed in resistant cell lines. SLC9B2 is under‐expressed in the TCR cell line and encodes for NHA2 protein that belongs to the sodium hydrogen antiporter family.^51^ Aside from being involved in cell pH and sodium regulation, it has also been reported that a downregulation of NHA2 protein could affect endocytosis^52^ and therefore could limit T‐DM1 internalization. Further studies are required to determine whether these SLCs are involved in T‐DM1 resistance mechanisms.

In conclusion the variants resistant to T‐DM1 did not display enhanced drug efflux while a decreased target antigen expression is not likely to be the main mechanism for drug resistance. Our results suggest that resistant cells possess an altered phenotype, including size and ploidy. Numerous alterations in SLC transporters, including several reported to be contained in lysosomes, were observed, warranting additional investigations of these transporters in the determination of resistance to T‐DM1.

## CONFLICT OF INTEREST

CD received research funding from Roche France. JS, LC, SB, KC, LJ, and EM have no conflict of interest.

## AUTHORS’ CONTRIBUTIONS

JS, LC, and CD designed and performed experiments, interpreted results, and wrote the manuscript. SB and ELM designed and performed experiments. LPJ and KC designed experiments and interpreted results. All authors have read and approved the manuscript.

## ETHICAL STATEMENT

Ethical statement is not required.

## Supporting information

Table S1‐Figure S1‐Figure S2

## Data Availability

The data that support the findings of this study are available from the corresponding author upon reasonable request.

## References

[1] Slamon DJ , Clark GM , Wong SG , Levin WJ , Ullrich A , McGuire WL . Human breast cancer: correlation of relapse and survival with amplification of the HER‐2/neu oncogene. Science. 1987;235:177‐182. 3798106 10.1126/science.3798106

[2] Iqbal N , Iqbal N . Human epidermal growth factor receptor 2 (HER2) in cancers: overexpression and therapeutic implications. Mol Biol Int. 2014;2014:852748. 25276427 10.1155/2014/852748 PMC4170925

[3] Sareyeldin RM , Gupta I , Al‐Hashimi I , et al. Expression and miRNAs profiling: function and regulation in human epidermal growth factor receptor 2 (HER2)‐ positive breast cancer. Cancers. 2019;11:646. 10.3390/cancers11050646 PMC6562440 31083383

[4] Dean L .Trastuzumab (Herceptin) therapy and ERBB2 (HER2) genotype In: Pratt V, McLeod H, Rubinstein W, Dean L, Kattman B, Malheiro A, eds. Medical genetics summaries. Bethesda, MD: National Center for Biotechnology Information (US); 2012. 28520340

[5] Hamy AS , Belin L , Bonsang‐Kitzis H , et al. Pathological complete response and prognosis after neoadjuvant chemotherapy for HER2‐positive breast cancers before and after trastuzumab era: results from a real‐life cohort. Br J Cancer. 2016;114:44‐52. 26657653 10.1038/bjc.2015.426 PMC4716543

[6] Loibl S , Gianni L . HER2‐positive breast cancer. Lancet. 2017;389:2415‐2429. 27939064 10.1016/S0140-6736(16)32417-5

[7] Reichert JM . Monoclonal antibodies in the clinic. Nat Biotechnol. 2001;19:819‐822. 11533635 10.1038/nbt0901-819

[8] Gerratana L , Bonotto M , Bozza C , et al. Pertuzumab and breast cancer: another piece in the anti‐HER2 puzzle. Expert Opin Biol Ther. 2017;17:365‐374. 28092723 10.1080/14712598.2017.1282944

[9] Junttila TT , Li G , Parsons K , Phillips GL , Sliwkowski MX . Trastuzumab‐DM1 (T‐DM1) retains all the mechanisms of action of trastuzumab and efficiently inhibits growth of lapatinib insensitive breast cancer. Breast Cancer Res Treat. 2011;128:347‐356. 20730488 10.1007/s10549-010-1090-x

[10] Lambert JM . Drug‐conjugated antibodies for the treatment of cancer. Br J Clin Pharmacol. 2013;76:248‐262. 23173552 10.1111/bcp.12044 PMC3731599

[11] Martinez MT , Perez‐Fidalgo JA , Martin‐Martorell P , et al. Treatment of HER2 positive advanced breast cancer with T‐DM1: a review of the literature. Crit Rev Oncol/Hematol. 2016;97:96‐106. 10.1016/j.critrevonc.2015.08.011 26318092

[12] Moek KL , de Groot DJA , de Vries EGE , Fehrmann RSN . The antibody‐drug conjugate target landscape across a broad range of tumour types. Ann Oncol. 2017;28:3083‐3091. 29045509 10.1093/annonc/mdx541

[13] von Minckwitz G , Huang CS , Mano MS , et al. Trastuzumab emtansine for residual invasive HER2‐positive breast cancer. N Engl J Med. 2019;380:617‐628. 30516102 10.1056/NEJMoa1814017

[14] Lambert JM , Chari RV . Ado‐trastuzumab Emtansine (T‐DM1): an antibody‐drug conjugate (ADC) for HER2‐positive breast cancer. J Med Chem. 2014;57:6949‐6964. 24967516 10.1021/jm500766w

[15] Collins DM , Bossenmaier B , Kollmorgen G , Niederfellner G . Acquired resistance to antibody‐drug conjugates. Cancers. 2019;11:394. 10.3390/cancers11030394 PMC6468698 30897808

[16] Nagy P , Friedlander E , Tanner M , et al. Decreased accessibility and lack of activation of ErbB2 in JIMT‐1, a herceptin‐resistant, MUC4‐expressing breast cancer cell line. Can Res. 2005;65:473‐482. 15695389

[17] Arribas J , Baselga J , Pedersen K , Parra‐Palau JL . p95HER2 and breast cancer. Can Res. 2011;71:1515‐1519. 10.1158/0008-5472.CAN-10-3795 21343397

[18] Barok M , Joensuu H , Isola J . Trastuzumab emtansine: mechanisms of action and drug resistance. Breast Cancer Res 2014;16:209. 24887180 10.1186/bcr3621 PMC4058749

[19] Chen R , Hou J , Newman E , et al. CD30 downregulation, MMAE resistance, and MDR1 upregulation are all associated with resistance to brentuximab vedotin. Mol Cancer Ther. 2015;14:1376‐1384. 25840583 10.1158/1535-7163.MCT-15-0036 PMC4458438

[20] Loganzo F , Tan X , Sung M , et al. Tumor cells chronically treated with a trastuzumab‐maytansinoid antibody‐drug conjugate develop varied resistance mechanisms but respond to alternate treatments. Mol Cancer Ther. 2015;14:952‐963. 25646013 10.1158/1535-7163.MCT-14-0862

[21] Loganzo F , Sung M , Gerber HP . Mechanisms of resistance to antibody‐drug conjugates. Mol Cancer Ther. 2016;15:2825‐2834. 27780876 10.1158/1535-7163.MCT-16-0408

[22] Kovtun YV , Goldmacher VS . Cell killing by antibody‐drug conjugates. Cancer Lett. 2007;255:232‐240. 17553616 10.1016/j.canlet.2007.04.010

[23] Hamblett KJ , Jacob AP , Gurgel JL , et al. SLC46A3 is required to transport catabolites of noncleavable antibody maytansine conjugates from the lysosome to the cytoplasm. Can Res. 2015;75:5329‐5340. 10.1158/0008-5472.CAN-15-1610 26631267

[24] Rai A , Kapoor S , Naaz A , Kumar Santra M , Panda D . Enhanced stability of microtubules contributes in the development of colchicine resistance in MCF‐7 cells. Biochem Pharmacol. 2017;132:38‐47. 28242250 10.1016/j.bcp.2017.02.018

[25] Dumontet C , Jordan MA . Microtubule‐binding agents: a dynamic field of cancer therapeutics. Nat Rev Drug Discov. 2010;9:790‐803. 20885410 10.1038/nrd3253 PMC3194401

[26] Webb M , Raphael CL , Asbahr H , Erber WN , Meyer BF . The detection of rhodamine 123 efflux at low levels of drug resistance. Br J Haematol. 1996;93:650‐655. 8652387 10.1046/j.1365-2141.1996.d01-1680.x

[27] Takara K , Sakaeda T , Okumura K . An update on overcoming MDR1‐mediated multidrug resistance in cancer chemotherapy. Curr Pharm Des. 2006;12:273‐286. 16454744 10.2174/138161206775201965

[28] Wu Q , Yang Z , Nie Y , Shi Y , Fan D . Multi‐drug resistance in cancer chemotherapeutics: mechanisms and lab approaches. Cancer Lett. 2014;347:159‐166. 24657660 10.1016/j.canlet.2014.03.013

[29] Gottesman MM , Fojo T , Bates SE . Multidrug resistance in cancer: role of ATP‐dependent transporters. Nat Rev Cancer. 2002;2:48‐58. 11902585 10.1038/nrc706

[30] Chen Z , Shi T , Zhang L , et al. Mammalian drug efflux transporters of the ATP binding cassette (ABC) family in multidrug resistance: a review of the past decade. Cancer Lett. 2016;370:153‐164. 26499806 10.1016/j.canlet.2015.10.010

[31] Dlugosz A , Janecka A . ABC transporters in the development of multidrug resistance in cancer therapy. Curr Pharm Des. 2016;22:4705‐4716. 26932159 10.2174/1381612822666160302103646

[32] Ejendal KF , Hrycyna CA . Differential sensitivities of the human ATP‐binding cassette transporters ABCG2 and P‐glycoprotein to cyclosporin A. Mol Pharmacol. 2005;67:902‐911. 15598974 10.1124/mol.104.001701

[33] Xia CQ , Liu N , Miwa GT , Gan LS . Interactions of cyclosporin a with breast cancer resistance protein. Drug Metab Dispos. 2007;35:576‐582. 17220244 10.1124/dmd.106.011866

[34] Mossink MH , van Zon A , Scheper RJ , Sonneveld P , Wiemer EA . Vaults: a ribonucleoprotein particle involved in drug resistance? Oncogene. 2003;22:7458‐7467. 14576851 10.1038/sj.onc.1206947

[35] Scheffer GL , Schroeijers AB , Izquierdo MA , Wiemer EA , Scheper RJ . Lung resistance‐related protein/major vault protein and vaults in multidrug‐resistant cancer. Curr Opin Oncol. 2000;12:550‐556. 11085454 10.1097/00001622-200011000-00007

[36] Lu JF , Pokharel D , Bebawy M . MRP1 and its role in anticancer drug resistance. Drug Metab Rev. 2015;47:406‐419. 26541366 10.3109/03602532.2015.1105253

[37] Verma S , Miles D , Gianni L , et al. Trastuzumab emtansine for HER2‐positive advanced breast cancer. N Engl J Med. 2012;367:1783‐1791. 23020162 10.1056/NEJMoa1209124 PMC5125250

[38] Hurvitz SA , Dirix L , Kocsis J , et al. Phase II randomized study of trastuzumab emtansine versus trastuzumab plus docetaxel in patients with human epidermal growth factor receptor 2‐positive metastatic breast cancer. J Clin Oncol. 2013;31:1157‐1163. 23382472 10.1200/JCO.2012.44.9694

[39] Kavallaris M . Microtubules and resistance to tubulin‐binding agents. Nat Rev Cancer. 2010;10:194‐204. 20147901 10.1038/nrc2803

[40] Gordon DJ , Resio B , Pellman D . Causes and consequences of aneuploidy in cancer. Nat Rev Genet. 2012;13:189‐203. 22269907 10.1038/nrg3123

[41] Sansregret L , Vanhaesebroeck B , Swanton C . Determinants and clinical implications of chromosomal instability in cancer. Nat Rev Clin Oncol. 2018;15:139‐150. 29297505 10.1038/nrclinonc.2017.198

[42] Byrd JC , Furman RR , Coutre SE , et al. Three‐year follow‐up of treatment‐naive and previously treated patients with CLL and SLL receiving single‐agent ibrutinib. Blood. 2015;125:2497‐2506. 25700432 10.1182/blood-2014-10-606038 PMC4400288

[43] Byrd JC , Furman RR , Coutre SE , et al. Targeting BTK with ibrutinib in relapsed chronic lymphocytic leukemia. N Engl J Med. 2013;369:32‐42. 23782158 10.1056/NEJMoa1215637 PMC3772525

[44] Davoli T , Uno H , Wooten EC , Elledge SJ . Tumor aneuploidy correlates with markers of immune evasion and with reduced response to immunotherapy. Science. 2017;355(6322):eaaf8399 28104840 10.1126/science.aaf8399 PMC5592794

[45] Al‐Abdulla R , Perez‐Silva L , Abete L , Romero MR , Briz O , Marin JJG . Unraveling 'The Cancer Genome Atlas' information on the role of SLC transporters in anticancer drug uptake. Expert Rev Clin Pharmacol. 2019;12:329‐341. 30744443 10.1080/17512433.2019.1581605

[46] Lin L , Yee SW , Kim RB , Giacomini KM . SLC transporters as therapeutic targets: emerging opportunities. Nat Rev Drug Discov. 2015;14:543‐560. 26111766 10.1038/nrd4626 PMC4698371

[47] Li Q , Shu Y . Role of solute carriers in response to anticancer drugs. Mol Cell Ther. 2014;2:15. 26056583 10.1186/2052-8426-2-15 PMC4452062

[48] Kinneer K , Meekin J , Tiberghien AC , et al. SLC46A3 as a potential predictive biomarker for antibody‐drug conjugates bearing noncleavable linked maytansinoid and pyrrolobenzodiazepine warheads. Clin Cancer Res. 2018;24:6570‐6582. 30131388 10.1158/1078-0432.CCR-18-1300

[49] Kukic I , Kelleher SL , Kiselyov K . Zn2+ efflux through lysosomal exocytosis prevents Zn2+‐induced toxicity. J Cell Sci. 2014;127(Pt 14):3094‐3103. 24829149 10.1242/jcs.145318 PMC4095854

[50] Koh JY , Kim HN , Hwang JJ , Kim YH , Park SE . Lysosomal dysfunction in proteinopathic neurodegenerative disorders: possible therapeutic roles of cAMP and zinc. Molecular Brain. 2019;12:18. 30866990 10.1186/s13041-019-0439-2 PMC6417073

[51] Xiang M , Feng M , Muend S , Rao R . A human Na+/H+ antiporter sharing evolutionary origins with bacterial NhaA may be a candidate gene for essential hypertension. Proc Natl Acad Sci USA. 2007;104:18677‐18681. 18000046 10.1073/pnas.0707120104 PMC2141836

[52] Deisl C , Simonin A , Anderegg M , et al. Sodium/hydrogen exchanger NHA2 is critical for insulin secretion in beta‐cells. Proc Natl Acad Sci USA. 2013;110:10004‐10009. 23720317 10.1073/pnas.1220009110 PMC3683798

