# Supplementary material for: Characterization of T‐DM1‐resistant breast cancer cells
Source: Pharmacol Res Perspect. 2020 Jun 24;8(4):e00617. doi: 10.1002/prp2.617 (PMC7314699; doi:10.1002/prp2.617)
Supplement: Supplementary file 1 — Table S1‐Figure S1‐Figure S2 [file PRP2-8-e00617-s001.pptx]

## Slide 1
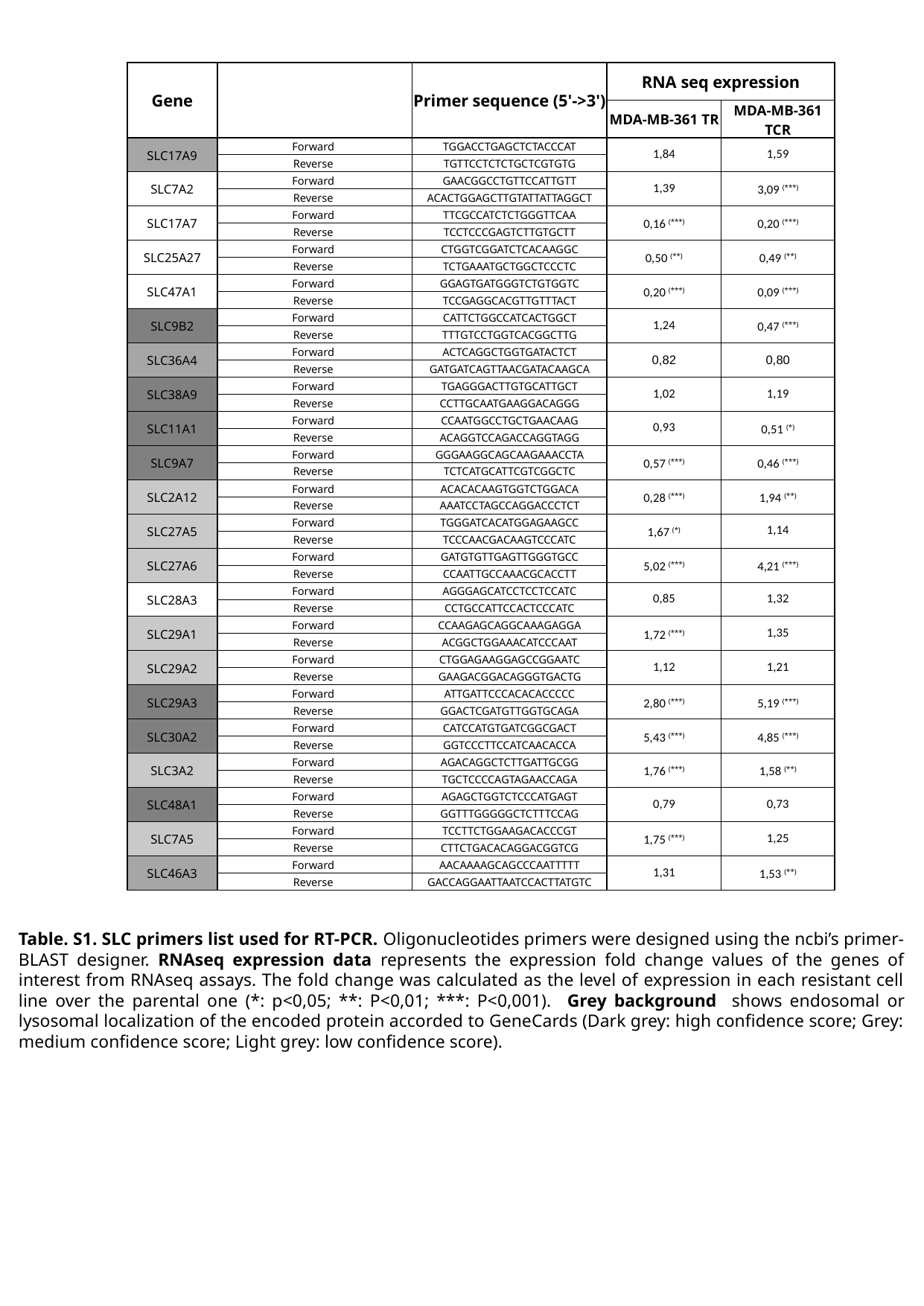

| Gene | | Primer sequence (5'->3') | RNA seq expression | |
| --- | --- | --- | --- | --- |
| | | | MDA-MB-361 TR | MDA-MB-361 TCR |
| SLC17A9 | Forward | TGGACCTGAGCTCTACCCAT | 1,84 | 1,59 |
| | Reverse | TGTTCCTCTCTGCTCGTGTG | | |
| SLC7A2 | Forward | GAACGGCCTGTTCCATTGTT | 1,39 | 3,09 (\*\*\*) |
| | Reverse | ACACTGGAGCTTGTATTATTAGGCT | | |
| SLC17A7 | Forward | TTCGCCATCTCTGGGTTCAA | 0,16 (\*\*\*) | 0,20 (\*\*\*) |
| | Reverse | TCCTCCCGAGTCTTGTGCTT | | |
| SLC25A27 | Forward | CTGGTCGGATCTCACAAGGC | 0,50 (\*\*) | 0,49 (\*\*) |
| | Reverse | TCTGAAATGCTGGCTCCCTC | | |
| SLC47A1 | Forward | GGAGTGATGGGTCTGTGGTC | 0,20 (\*\*\*) | 0,09 (\*\*\*) |
| | Reverse | TCCGAGGCACGTTGTTTACT | | |
| SLC9B2 | Forward | CATTCTGGCCATCACTGGCT | 1,24 | 0,47 (\*\*\*) |
| | Reverse | TTTGTCCTGGTCACGGCTTG | | |
| SLC36A4 | Forward | ACTCAGGCTGGTGATACTCT | 0,82 | 0,80 |
| | Reverse | GATGATCAGTTAACGATACAAGCA | | |
| SLC38A9 | Forward | TGAGGGACTTGTGCATTGCT | 1,02 | 1,19 |
| | Reverse | CCTTGCAATGAAGGACAGGG | | |
| SLC11A1 | Forward | CCAATGGCCTGCTGAACAAG | 0,93 | 0,51 (\*) |
| | Reverse | ACAGGTCCAGACCAGGTAGG | | |
| SLC9A7 | Forward | GGGAAGGCAGCAAGAAACCTA | 0,57 (\*\*\*) | 0,46 (\*\*\*) |
| | Reverse | TCTCATGCATTCGTCGGCTC | | |
| SLC2A12 | Forward | ACACACAAGTGGTCTGGACA | 0,28 (\*\*\*) | 1,94 (\*\*) |
| | Reverse | AAATCCTAGCCAGGACCCTCT | | |
| SLC27A5 | Forward | TGGGATCACATGGAGAAGCC | 1,67 (\*) | 1,14 |
| | Reverse | TCCCAACGACAAGTCCCATC | | |
| SLC27A6 | Forward | GATGTGTTGAGTTGGGTGCC | 5,02 (\*\*\*) | 4,21 (\*\*\*) |
| | Reverse | CCAATTGCCAAACGCACCTT | | |
| SLC28A3 | Forward | AGGGAGCATCCTCCTCCATC | 0,85 | 1,32 |
| | Reverse | CCTGCCATTCCACTCCCATC | | |
| SLC29A1 | Forward | CCAAGAGCAGGCAAAGAGGA | 1,72 (\*\*\*) | 1,35 |
| | Reverse | ACGGCTGGAAACATCCCAAT | | |
| SLC29A2 | Forward | CTGGAGAAGGAGCCGGAATC | 1,12 | 1,21 |
| | Reverse | GAAGACGGACAGGGTGACTG | | |
| SLC29A3 | Forward | ATTGATTCCCACACACCCCC | 2,80 (\*\*\*) | 5,19 (\*\*\*) |
| | Reverse | GGACTCGATGTTGGTGCAGA | | |
| SLC30A2 | Forward | CATCCATGTGATCGGCGACT | 5,43 (\*\*\*) | 4,85 (\*\*\*) |
| | Reverse | GGTCCCTTCCATCAACACCA | | |
| SLC3A2 | Forward | AGACAGGCTCTTGATTGCGG | 1,76 (\*\*\*) | 1,58 (\*\*) |
| | Reverse | TGCTCCCCAGTAGAACCAGA | | |
| SLC48A1 | Forward | AGAGCTGGTCTCCCATGAGT | 0,79 | 0,73 |
| | Reverse | GGTTTGGGGGCTCTTTCCAG | | |
| SLC7A5 | Forward | TCCTTCTGGAAGACACCCGT | 1,75 (\*\*\*) | 1,25 |
| | Reverse | CTTCTGACACAGGACGGTCG | | |
| SLC46A3 | Forward | AACAAAAGCAGCCCAATTTTT | 1,31 | 1,53 (\*\*) |
| | Reverse | GACCAGGAATTAATCCACTTATGTC | | |
Table. S1. SLC primers list used for RT-PCR. Oligonucleotides primers were designed using the ncbi’s primer-BLAST designer. RNAseq expression data represents the expression fold change values of the genes of interest from RNAseq assays. The fold change was calculated as the level of expression in each resistant cell line over the parental one (*: p<0,05; **: P<0,01; ***: P<0,001). Grey background shows endosomal or lysosomal localization of the encoded protein accorded to GeneCards (Dark grey: high confidence score; Grey: medium confidence score; Light grey: low confidence score).

## Slide 2
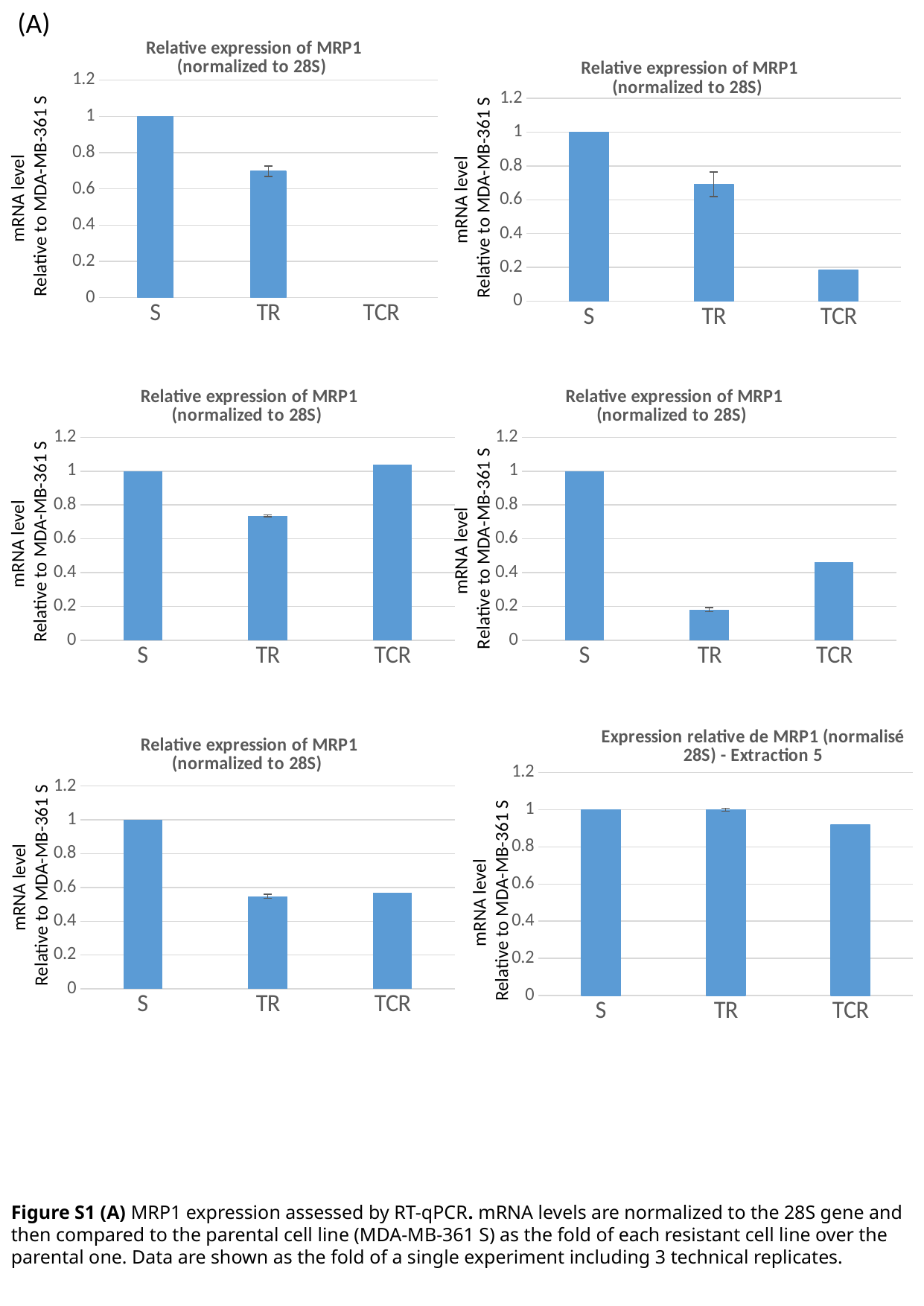

(A)
[unsupported chart]
### Chart: Relative expression of MRP1 (normalized to 28S)
| Category | S |
|---|---|
| S | 1.0 |
| TR | 0.6925547340554565 |
| TCR | 0.18258835557625863 |mRNA level
Relative to MDA-MB-361 S
mRNA level
Relative to MDA-MB-361 S
### Chart: Relative expression of MRP1 (normalized to 28S)
| Category | S |
|---|---|
| S | 1.0 |
| TR | 0.7354334320929758 |
| TCR | 1.0400599338884815 |
### Chart: Relative expression of MRP1 (normalized to 28S)
| Category | S |
|---|---|
| S | 1.0 |
| TR | 0.17965902732236605 |
| TCR | 0.4600938253124383 |mRNA level
Relative to MDA-MB-361 S
mRNA level
Relative to MDA-MB-361 S
### Chart: Expression relative de MRP1 (normalisé 28S) - Extraction 5
| Category | S |
|---|---|
| S | 1.0 |
| TR | 1.0 |
| TCR | 0.9180640199652165 |
### Chart: Relative expression of MRP1 (normalized to 28S)
| Category | S |
|---|---|
| S | 1.0 |
| TR | 0.5484124898473128 |
| TCR | 0.5690655172939101 |mRNA level
Relative to MDA-MB-361 S
mRNA level
Relative to MDA-MB-361 S
Figure S1 (A) MRP1 expression assessed by RT-qPCR. mRNA levels are normalized to the 28S gene and then compared to the parental cell line (MDA-MB-361 S) as the fold of each resistant cell line over the parental one. Data are shown as the fold of a single experiment including 3 technical replicates.

## Slide 3
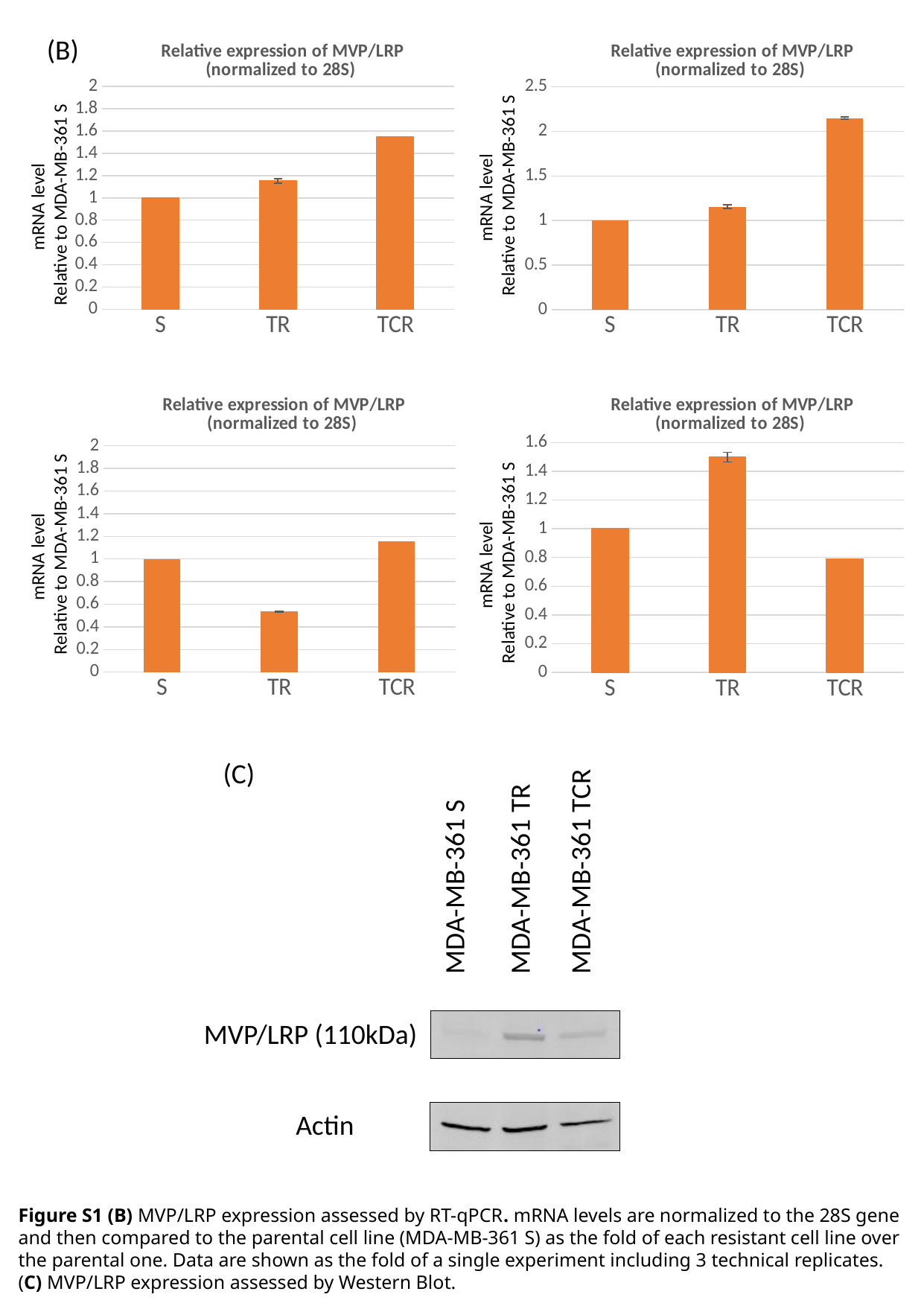

(B)
### Chart: Relative expression of MVP/LRP (normalized to 28S)
| Category | S |
|---|---|
| S | 1.0 |
| TR | 1.154018751763562 |
| TCR | 1.5475649935423925 |
### Chart: Relative expression of MVP/LRP (normalized to 28S)
| Category | S |
|---|---|
| S | 1.0 |
| TR | 1.1566881839052796 |
| TCR | 2.1509887809147408 |mRNA level
Relative to MDA-MB-361 S
mRNA level
Relative to MDA-MB-361 S
### Chart: Relative expression of MVP/LRP (normalized to 28S)
| Category | S |
|---|---|
| S | 1.0 |
| TR | 0.5346499992908696 |
| TCR | 1.1540187517635534 |
### Chart: Relative expression of MVP/LRP (normalized to 28S)
| Category | S |
|---|---|
| S | 1.0 |
| TR | 1.4983070768766842 |
| TCR | 0.7900413118633753 |mRNA level
Relative to MDA-MB-361 S
mRNA level
Relative to MDA-MB-361 S
(C)
MDA-MB-361 TCR
MDA-MB-361 TR
MDA-MB-361 S
MVP/LRP (110kDa)
Actin
Figure S1 (B) MVP/LRP expression assessed by RT-qPCR. mRNA levels are normalized to the 28S gene and then compared to the parental cell line (MDA-MB-361 S) as the fold of each resistant cell line over the parental one. Data are shown as the fold of a single experiment including 3 technical replicates. (C) MVP/LRP expression assessed by Western Blot.

## Slide 4
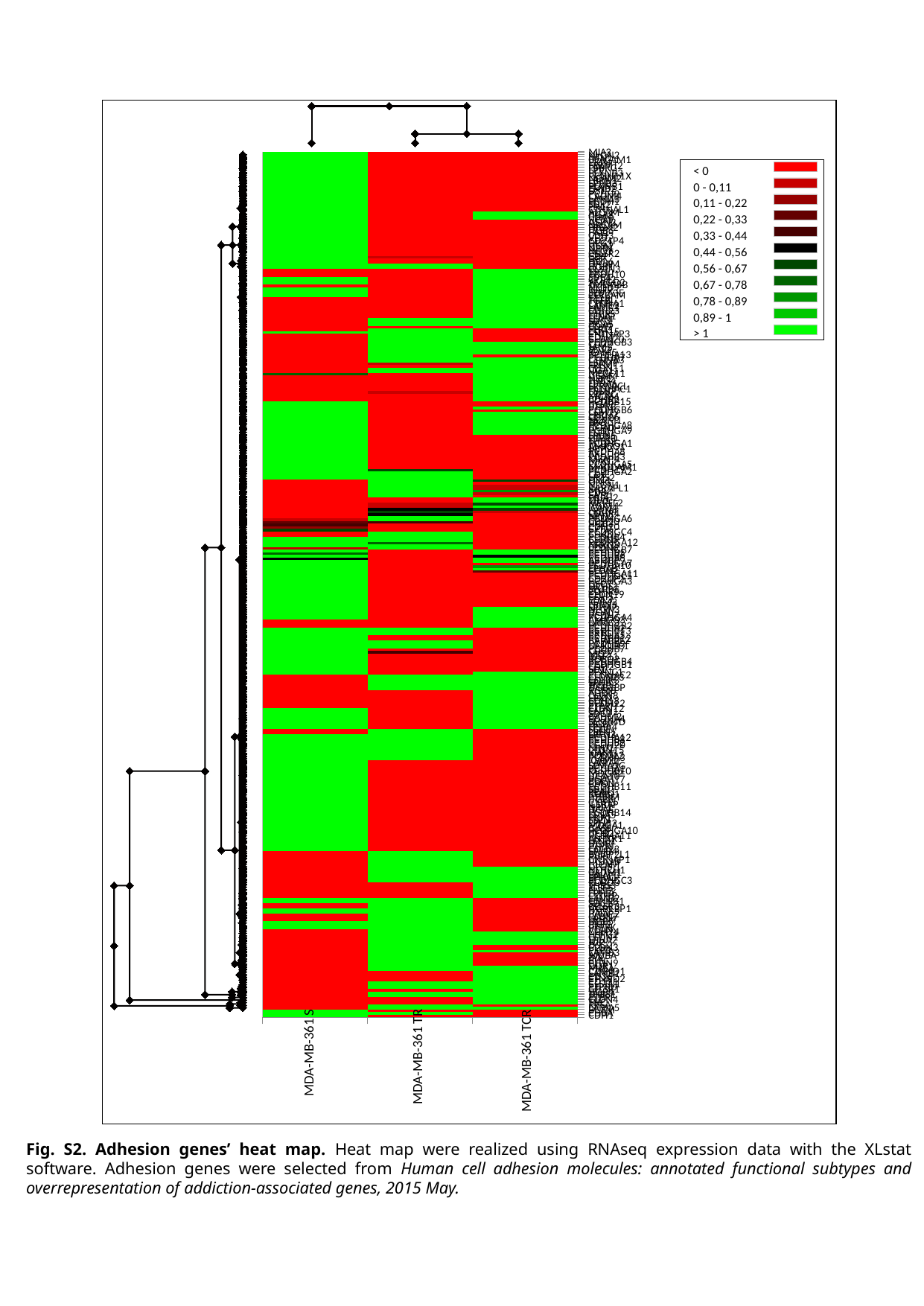

### Chart
| Category | |
|---|---|
### Chart
| Category | |
|---|---|
### Chart
| Category | | | |
|---|---|---|---|
| CDH1 | 1.0 | 1.0 | 1.0 |
| CD8A | 1.0 | 1.0 | 1.0 |
| BCAM | 1.0 | 1.0 | 1.0 |
| LAMA5 | 1.0 | 1.0 | 1.0 |
| RPSA | 1.0 | 1.0 | 1.0 |
| TNC | 1.0 | 1.0 | 1.0 |
| CLDN4 | 1.0 | 1.0 | 1.0 |
| PTPRF | 1.0 | 1.0 | 1.0 |
| THBS1 | 1.0 | 1.0 | 1.0 |
| ITGB4 | 1.0 | 1.0 | 1.0 |
| CELSR1 | 1.0 | 1.0 | 1.0 |
| PTPRU | 1.0 | 1.0 | 1.0 |
| CLSTN1 | 1.0 | 1.0 | 1.0 |
| CD151 | 1.0 | 1.0 | 1.0 |
| CTNND2 | 1.0 | 1.0 | 1.0 |
| CD36 | 1.0 | 1.0 | 1.0 |
| LAMB1 | 1.0 | 1.0 | 1.0 |
| CTNND1 | 1.0 | 1.0 | 1.0 |
| MAEA | 1.0 | 1.0 | 1.0 |
| DDR1 | 1.0 | 1.0 | 1.0 |
| CLDN9 | 1.0 | 1.0 | 1.0 |
| BYSL | 1.0 | 1.0 | 1.0 |
| ZYX | 1.0 | 1.0 | 1.0 |
| AJUBA | 1.0 | 1.0 | 1.0 |
| LAMB3 | 1.0 | 1.0 | 1.0 |
| PKP3 | 1.0 | 1.0 | 1.0 |
| CLDN3 | 1.0 | 1.0 | 1.0 |
| DSG2 | 1.0 | 1.0 | 1.0 |
| JUP | 1.0 | 1.0 | 1.0 |
| EPHA2 | 1.0 | 1.0 | 1.0 |
| CLDN1 | 1.0 | 1.0 | 1.0 |
| HSPG2 | 1.0 | 1.0 | 1.0 |
| CDH24 | 1.0 | 1.0 | 1.0 |
| VCAN | 1.0 | 1.0 | 1.0 |
| PTPRK | 1.0 | 1.0 | 1.0 |
| PKP2 | 1.0 | 1.0 | 1.0 |
| ITGB5 | 1.0 | 1.0 | 1.0 |
| LRFN4 | 1.0 | 1.0 | 1.0 |
| CLDN7 | 1.0 | 1.0 | 1.0 |
| BAIAP2 | 1.0 | 1.0 | 1.0 |
| DSC2 | 1.0 | 1.0 | 1.0 |
| ITGB1BP1 | 1.0 | 1.0 | 1.0 |
| CELSR3 | 1.0 | 1.0 | 1.0 |
| SDC1 | 1.0 | 1.0 | 1.0 |
| CTNNB1 | 1.0 | 1.0 | 1.0 |
| LAMB2 | 1.0 | 1.0 | 1.0 |
| CYTH1 | 1.0 | 1.0 | 1.0 |
| EPHB6 | 1.0 | 1.0 | 1.0 |
| FLRT3 | 1.0 | 1.0 | 1.0 |
| IGSF5 | 1.0 | 1.0 | 1.0 |
| THBS3 | 1.0 | 1.0 | 1.0 |
| ROBO3 | 1.0 | 1.0 | 1.0 |
| PCDHGC3 | 1.0 | 1.0 | 1.0 |
| EPHA1 | 1.0 | 1.0 | 1.0 |
| BAIAP3 | 1.0 | 1.0 | 1.0 |
| CADM1 | 1.0 | 1.0 | 1.0 |
| NOTCH1 | 1.0 | 1.0 | 1.0 |
| DLG5 | 1.0 | 1.0 | 1.0 |
| L1CAM | 1.0 | 1.0 | 1.0 |
| ITGA2B | 1.0 | 1.0 | 1.0 |
| CNTNAP1 | 1.0 | 1.0 | 1.0 |
| PVR | 1.0 | 1.0 | 1.0 |
| BAIAP2L1 | 1.0 | 1.0 | 1.0 |
| EPHB4 | 1.0 | 1.0 | 1.0 |
| CDH13 | 1.0 | 1.0 | 1.0 |
| PTPRJ | 1.0 | 1.0 | 1.0 |
| ITGA7 | 1.0 | 1.0 | 1.0 |
| EFNB1 | 1.0 | 1.0 | 1.0 |
| ANTXR1 | 1.0 | 1.0 | 1.0 |
| PCDHA11 | 1.0 | 1.0 | 1.0 |
| ITGB2 | 1.0 | 1.0 | 1.0 |
| PCDHGA10 | 1.0 | 1.0 | 1.0 |
| GAS6 | 1.0 | 1.0 | 1.0 |
| MDGA1 | 1.0 | 1.0 | 1.0 |
| EPHB2 | 1.0 | 1.0 | 1.0 |
| SSPN | 1.0 | 1.0 | 1.0 |
| PKP1 | 1.0 | 1.0 | 1.0 |
| EFNA5 | 1.0 | 1.0 | 1.0 |
| PCDHB14 | 1.0 | 1.0 | 1.0 |
| ITGAE | 1.0 | 1.0 | 1.0 |
| NRP1 | 1.0 | 1.0 | 1.0 |
| IL1RAP | 1.0 | 1.0 | 1.0 |
| CYTH3 | 1.0 | 1.0 | 1.0 |
| ITGB8 | 1.0 | 1.0 | 1.0 |
| PTPRM | 1.0 | 1.0 | 1.0 |
| ROBO1 | 1.0 | 1.0 | 1.0 |
| PEAK1 | 1.0 | 1.0 | 1.0 |
| SDK1 | 1.0 | 1.0 | 1.0 |
| PCDHB11 | 1.0 | 1.0 | 1.0 |
| CDON | 1.0 | 1.0 | 1.0 |
| BOC | 1.0 | 1.0 | 1.0 |
| PCDH17 | 1.0 | 1.0 | 1.0 |
| ITGA10 | 1.0 | 1.0 | 1.0 |
| MDGA2 | 1.0 | 1.0 | 1.0 |
| PCDHB10 | 1.0 | 1.0 | 1.0 |
| PCDHA1 | 1.0 | 1.0 | 1.0 |
| SEMA3G | 1.0 | 1.0 | 1.0 |
| LAMA4 | 1.0 | 1.0 | 1.0 |
| ICAM1 | 1.0 | 1.0 | 1.0 |
| PCDHA3 | 1.0 | 1.0 | 1.0 |
| PODXL2 | 1.0 | 1.0 | 1.0 |
| ICAM3 | 1.0 | 1.0 | 1.0 |
| CLDN15 | 1.0 | 1.0 | 1.0 |
| NINJ1 | 1.0 | 1.0 | 1.0 |
| PCDH20 | 1.0 | 1.0 | 1.0 |
| PCDHB2 | 1.0 | 1.0 | 1.0 |
| PCDHB8 | 1.0 | 1.0 | 1.0 |
| PCDHA12 | 1.0 | 1.0 | 1.0 |
| LRFN3 | 1.0 | 1.0 | 1.0 |
| LRFN1 | 1.0 | 1.0 | 1.0 |
| SGCE | 1.0 | 1.0 | 1.0 |
| EPHA4 | 1.0 | 1.0 | 1.0 |
| ITGB7 | 1.0 | 1.0 | 1.0 |
| SEMA4D | 1.0 | 1.0 | 1.0 |
| PCDHA4 | 1.0 | 1.0 | 1.0 |
| CADM2 | 1.0 | 1.0 | 1.0 |
| SDC3 | 1.0 | 1.0 | 1.0 |
| CNTN1 | 1.0 | 1.0 | 1.0 |
| CLDN12 | 1.0 | 1.0 | 1.0 |
| PTPRH | 1.0 | 1.0 | 1.0 |
| PCDH12 | 1.0 | 1.0 | 1.0 |
| CDH18 | 1.0 | 1.0 | 1.0 |
| LPXN | 1.0 | 1.0 | 1.0 |
| CLDN8 | 1.0 | 1.0 | 1.0 |
| AGER | 1.0 | 1.0 | 1.0 |
| RGMB | 1.0 | 1.0 | 1.0 |
| ITGB3BP | 1.0 | 1.0 | 1.0 |
| TNXB | 1.0 | 1.0 | 1.0 |
| EPHA3 | 1.0 | 1.0 | 1.0 |
| CDHR3 | 1.0 | 1.0 | 1.0 |
| CLDN23 | 1.0 | 1.0 | 1.0 |
| PCDHAC2 | 1.0 | 1.0 | 1.0 |
| PLXNC1 | 1.0 | 1.0 | 1.0 |
| SELL | 1.0 | 1.0 | 1.0 |
| CDH2 | 1.0 | 1.0 | 1.0 |
| PCDHGB1 | 1.0 | 1.0 | 1.0 |
| PCDHGB4 | 1.0 | 1.0 | 1.0 |
| ROBO2 | 1.0 | 1.0 | 1.0 |
| IGSF11 | 1.0 | 1.0 | 1.0 |
| NID2 | 1.0 | 1.0 | 1.0 |
| CD58 | 1.0 | 1.0 | 1.0 |
| PCDHB7 | 1.0 | 1.0 | 1.0 |
| UMODL1 | 1.0 | 1.0 | 1.0 |
| PCDHB9 | 1.0 | 1.0 | 1.0 |
| BAIAP2L2 | 1.0 | 1.0 | 1.0 |
| PCDHB12 | 1.0 | 1.0 | 1.0 |
| PCDHA5 | 1.0 | 1.0 | 1.0 |
| PRTG | 1.0 | 1.0 | 1.0 |
| PCDHB13 | 1.0 | 1.0 | 1.0 |
| PCDHA2 | 1.0 | 1.0 | 1.0 |
| PCDHGB2 | 1.0 | 1.0 | 1.0 |
| LAMC3 | 1.0 | 1.0 | 1.0 |
| AMIGO3 | 1.0 | 1.0 | 1.0 |
| PCDHGA4 | 1.0 | 1.0 | 1.0 |
| PCDH7 | 1.0 | 1.0 | 1.0 |
| ITGAL | 1.0 | 1.0 | 1.0 |
| NLGN3 | 1.0 | 1.0 | 1.0 |
| EPHA7 | 1.0 | 1.0 | 1.0 |
| LRRN3 | 1.0 | 1.0 | 1.0 |
| ICAM2 | 1.0 | 1.0 | 1.0 |
| EDIL3 | 1.0 | 1.0 | 1.0 |
| CDH5 | 1.0 | 1.0 | 1.0 |
| CLDN19 | 1.0 | 1.0 | 1.0 |
| CDHR5 | 1.0 | 1.0 | 1.0 |
| SELPLG | 1.0 | 1.0 | 1.0 |
| DCHS1 | 1.0 | 1.0 | 1.0 |
| DSC3 | 1.0 | 1.0 | 1.0 |
| PCDHGA3 | 1.0 | 1.0 | 1.0 |
| CD24P2 | 1.0 | 1.0 | 1.0 |
| PCDHGC5 | 1.0 | 1.0 | 1.0 |
| PCDHGA11 | 1.0 | 1.0 | 1.0 |
| CLDN2 | 1.0 | 1.0 | 1.0 |
| EPHA8 | 1.0 | 1.0 | 1.0 |
| PCDHA10 | 1.0 | 1.0 | 1.0 |
| PCDHGA7 | 1.0 | 1.0 | 1.0 |
| ABI3BP | 1.0 | 1.0 | 1.0 |
| PCDHA6 | 1.0 | 1.0 | 1.0 |
| PCDHB6 | 1.0 | 1.0 | 1.0 |
| PCDHB5 | 1.0 | 1.0 | 1.0 |
| PCDHGB7 | 1.0 | 1.0 | 1.0 |
| CLDN6 | 1.0 | 1.0 | 1.0 |
| NRXN3 | 1.0 | 1.0 | 1.0 |
| PCDHGA12 | 1.0 | 1.0 | 1.0 |
| CLDN5 | 1.0 | 1.0 | 1.0 |
| PCDHB4 | 1.0 | 1.0 | 1.0 |
| CD48 | 1.0 | 1.0 | 1.0 |
| PCDHGC4 | 1.0 | 1.0 | 1.0 |
| CYTIP | 1.0 | 1.0 | 1.0 |
| CDH10 | 1.0 | 1.0 | 1.0 |
| ITGB3 | 1.0 | 1.0 | 1.0 |
| CDH20 | 1.0 | 1.0 | 1.0 |
| PCDHGA6 | 1.0 | 1.0 | 1.0 |
| NINJ2 | 1.0 | 1.0 | 1.0 |
| CDHR4 | 1.0 | 1.0 | 1.0 |
| LRRN4 | 1.0 | 1.0 | 1.0 |
| ICAM4 | 1.0 | 1.0 | 1.0 |
| ITGA11 | 1.0 | 1.0 | 1.0 |
| KIRREL2 | 1.0 | 1.0 | 1.0 |
| THY1 | 1.0 | 1.0 | 1.0 |
| PRPH2 | 1.0 | 1.0 | 1.0 |
| FAT2 | 1.0 | 1.0 | 1.0 |
| CD84 | 1.0 | 1.0 | 1.0 |
| MPL | 1.0 | 1.0 | 1.0 |
| IL1RAPL1 | 1.0 | 1.0 | 1.0 |
| NLGN1 | 1.0 | 1.0 | 1.0 |
| OMG | 1.0 | 1.0 | 1.0 |
| DSC1 | 1.0 | 1.0 | 1.0 |
| LIMS2 | 1.0 | 1.0 | 1.0 |
| CD6 | 1.0 | 1.0 | 1.0 |
| PCDHGA2 | 1.0 | 1.0 | 1.0 |
| PCDH19 | 1.0 | 1.0 | 1.0 |
| MADCAM1 | 1.0 | 1.0 | 1.0 |
| PCDHGA5 | 1.0 | 1.0 | 1.0 |
| MAG | 1.0 | 1.0 | 1.0 |
| MFAP4 | 1.0 | 1.0 | 1.0 |
| PCDHB3 | 1.0 | 1.0 | 1.0 |
| AXL | 1.0 | 1.0 | 1.0 |
| PCDHA8 | 1.0 | 1.0 | 1.0 |
| JAM2 | 1.0 | 1.0 | 1.0 |
| AMIGO1 | 1.0 | 1.0 | 1.0 |
| PCDHGA1 | 1.0 | 1.0 | 1.0 |
| THBS2 | 1.0 | 1.0 | 1.0 |
| PTPRO | 1.0 | 1.0 | 1.0 |
| FBLN5 | 1.0 | 1.0 | 1.0 |
| ELFN1 | 1.0 | 1.0 | 1.0 |
| PCDHGA9 | 1.0 | 1.0 | 1.0 |
| ITGAD | 1.0 | 1.0 | 1.0 |
| PCDHGA8 | 1.0 | 1.0 | 1.0 |
| TRO | 1.0 | 1.0 | 1.0 |
| NCAM1 | 1.0 | 1.0 | 1.0 |
| CDH26 | 1.0 | 1.0 | 1.0 |
| LRRN2 | 1.0 | 1.0 | 1.0 |
| CDH17 | 1.0 | 1.0 | 1.0 |
| PCDHGB6 | 1.0 | 1.0 | 1.0 |
| PTPRB | 1.0 | 1.0 | 1.0 |
| ITGA9 | 1.0 | 1.0 | 1.0 |
| PCDHB15 | 1.0 | 1.0 | 1.0 |
| CDHR2 | 1.0 | 1.0 | 1.0 |
| MCAM | 1.0 | 1.0 | 1.0 |
| PTPRC | 1.0 | 1.0 | 1.0 |
| FBLN7 | 1.0 | 1.0 | 1.0 |
| PCDHAC1 | 1.0 | 1.0 | 1.0 |
| LRRN4CL | 1.0 | 1.0 | 1.0 |
| EPHA6 | 1.0 | 1.0 | 1.0 |
| THBS4 | 1.0 | 1.0 | 1.0 |
| NRP2 | 1.0 | 1.0 | 1.0 |
| ITGAM | 1.0 | 1.0 | 1.0 |
| MEGF11 | 1.0 | 1.0 | 1.0 |
| ITGA1 | 1.0 | 1.0 | 1.0 |
| CLDN11 | 1.0 | 1.0 | 1.0 |
| FREM1 | 1.0 | 1.0 | 1.0 |
| LSAMP | 1.0 | 1.0 | 1.0 |
| CTNNA3 | 1.0 | 1.0 | 1.0 |
| PCDHA7 | 1.0 | 1.0 | 1.0 |
| PCDHA13 | 1.0 | 1.0 | 1.0 |
| ICAM5 | 1.0 | 1.0 | 1.0 |
| SLIT2 | 1.0 | 1.0 | 1.0 |
| JAM3 | 1.0 | 1.0 | 1.0 |
| CD72 | 1.0 | 1.0 | 1.0 |
| PCDHGB3 | 1.0 | 1.0 | 1.0 |
| CLDN20 | 1.0 | 1.0 | 1.0 |
| CDH4 | 1.0 | 1.0 | 1.0 |
| CNTNAP3 | 1.0 | 1.0 | 1.0 |
| CDH15 | 1.0 | 1.0 | 1.0 |
| FLRT1 | 1.0 | 1.0 | 1.0 |
| CD47 | 1.0 | 1.0 | 1.0 |
| ITGA5 | 1.0 | 1.0 | 1.0 |
| SDC2 | 1.0 | 1.0 | 1.0 |
| ESAM | 1.0 | 1.0 | 1.0 |
| EFNA1 | 1.0 | 1.0 | 1.0 |
| PTPRS | 1.0 | 1.0 | 1.0 |
| CDH23 | 1.0 | 1.0 | 1.0 |
| LAMC1 | 1.0 | 1.0 | 1.0 |
| LAMC2 | 1.0 | 1.0 | 1.0 |
| CTNNA1 | 1.0 | 1.0 | 1.0 |
| TGFBI | 1.0 | 1.0 | 1.0 |
| F11R | 1.0 | 1.0 | 1.0 |
| CERCAM | 1.0 | 1.0 | 1.0 |
| SEMA3E | 1.0 | 1.0 | 1.0 |
| IGSF9 | 1.0 | 1.0 | 1.0 |
| NEDD9 | 1.0 | 1.0 | 1.0 |
| TMEM8B | 1.0 | 1.0 | 1.0 |
| AMIGO2 | 1.0 | 1.0 | 1.0 |
| CYTH2 | 1.0 | 1.0 | 1.0 |
| SDC4 | 1.0 | 1.0 | 1.0 |
| PCDH10 | 1.0 | 1.0 | 1.0 |
| TSC1 | 1.0 | 1.0 | 1.0 |
| CLSTN3 | 1.0 | 1.0 | 1.0 |
| ITGB1 | 1.0 | 1.0 | 1.0 |
| EPCAM | 1.0 | 1.0 | 1.0 |
| ITGA6 | 1.0 | 1.0 | 1.0 |
| DSP | 1.0 | 1.0 | 1.0 |
| CD9 | 1.0 | 1.0 | 1.0 |
| CELSR2 | 1.0 | 1.0 | 1.0 |
| NEO1 | 1.0 | 1.0 | 1.0 |
| NPTN | 1.0 | 1.0 | 1.0 |
| ITGA2 | 1.0 | 1.0 | 1.0 |
| DLG1 | 1.0 | 1.0 | 1.0 |
| CD24P4 | 1.0 | 1.0 | 1.0 |
| VEZT | 1.0 | 1.0 | 1.0 |
| CDH3 | 1.0 | 1.0 | 1.0 |
| FAT1 | 1.0 | 1.0 | 1.0 |
| ITGB6 | 1.0 | 1.0 | 1.0 |
| FREM2 | 1.0 | 1.0 | 1.0 |
| NRCAM | 1.0 | 1.0 | 1.0 |
| ASTN2 | 1.0 | 1.0 | 1.0 |
| ITGAV | 1.0 | 1.0 | 1.0 |
| CD44 | 1.0 | 1.0 | 1.0 |
| ITGA3 | 1.0 | 1.0 | 1.0 |
| ALCAM | 1.0 | 1.0 | 1.0 |
| CTNNAL1 | 1.0 | 1.0 | 1.0 |
| FN1 | 1.0 | 1.0 | 1.0 |
| SDK2 | 1.0 | 1.0 | 1.0 |
| PCDH1 | 1.0 | 1.0 | 1.0 |
| LAMA3 | 1.0 | 1.0 | 1.0 |
| CADM4 | 1.0 | 1.0 | 1.0 |
| PCDH9 | 1.0 | 1.0 | 1.0 |
| DST | 1.0 | 1.0 | 1.0 |
| EFNB2 | 1.0 | 1.0 | 1.0 |
| PLXNB1 | 1.0 | 1.0 | 1.0 |
| EPHB3 | 1.0 | 1.0 | 1.0 |
| LRRN1 | 1.0 | 1.0 | 1.0 |
| NCAM2 | 1.0 | 1.0 | 1.0 |
| PCDH11X | 1.0 | 1.0 | 1.0 |
| PLXNB3 | 1.0 | 1.0 | 1.0 |
| LPP | 1.0 | 1.0 | 1.0 |
| PTPRG | 1.0 | 1.0 | 1.0 |
| FERMT2 | 1.0 | 1.0 | 1.0 |
| PKP4 | 1.0 | 1.0 | 1.0 |
| CEACAM1 | 1.0 | 1.0 | 1.0 |
| NTN1 | 1.0 | 1.0 | 1.0 |
| NLGN2 | 1.0 | 1.0 | 1.0 |
| MIA3 | 1.0 | 1.0 | 1.0 | < 0
 0 - 0,11
 0,11 - 0,22
 0,22 - 0,33
 0,33 - 0,44
 0,44 - 0,56
 0,56 - 0,67
 0,67 - 0,78
 0,78 - 0,89
 0,89 - 1
 > 1
MDA-MB-361 S
MDA-MB-361 TR
MDA-MB-361 TCR
Fig. S2. Adhesion genes’ heat map. Heat map were realized using RNAseq expression data with the XLstat software. Adhesion genes were selected from Human cell adhesion molecules: annotated functional subtypes and overrepresentation of addiction-associated genes, 2015 May.
